# Why Hydrogen Dissociation Catalysts do not Work for Hydrogenation of Magnesium

**DOI:** 10.1002/advs.202304603

**Published:** 2023-12-09

**Authors:** Selim Kazaz, Emanuel Billeter, Filippo Longo, Andreas Borgschulte, Zbigniew Łodziana

**Affiliations:** ^1^ Laboratory for Advanced Analytical Technologies Swiss Federal Laboratories for Materials Science and Technology Empa Überlandstrasse 129 Dübendorf CH‐8600 Switzerland; ^2^ Department of Chemistry University of Zurich Winterthurerstrasse 190 Zürich CH‐8057 Switzerland; ^3^ Institute of Nuclear Physics Polish Academy of Sciences Krakow PL‐31342 Poland

**Keywords:** DFT, electron spectroscopy, hydrogen storage, hydrogenation catalyst, magnesium hydride, sorption kinetics

## Abstract

Provision of atomic hydrogen by hydrogen dissociation catalysts only moderately accelerates the hydrogenation rate of magnesium. They shed light on this well‐known but technically challenging fact through a combined approach using an unconventional surface science technique together with Density Functional Theory (DFT) calculations. The calculations demonstrate the drastic electronic structure changes during transformation of Mg to MgH_2_, which make fractional hydrogen coverage on the surface, as well as substoichiometric hydrogen content in the bulk energetically unfavorable. Reflecting Electron Energy Loss Spectroscopy (REELS) is used to measure the surface and bulk plasmon during hydrogen sorption in magnesium. The measurements show that the hydrogenation proceeds via the growth of magnesium hydride without the presence of chemisorbed hydrogen on the metallic magnesium surface exactly as indicated by the calculations. This is due to the low stability of sub‐stoichiometric amounts of chemisorbed H correlating with the unfavorable charge state of Mg. They are merely bound to the unchanged adjacent Mg layers, thereby explaining the failure of classical hydrogenation catalysts, which effectively only hydrogenate Mg in their direct vicinity. The acceleration of hydrogen sorption kinetics in Mg must affect the polarization in the interface between Mg and MgH_2_ during hydrogenation.

## Graphical abstract

Drastic electronic structure changes during the transformation of the surface and bulk of Mg to MgH_2_ as deduced from density functional theory calculations and in situ reflecting electron energy loss spectroscopy explain why hydrogen dissociation catalysts hardly accelerate the hydrogen sorption kinetics in Mg.

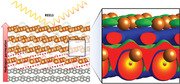

## 1 Introduction

Ionic hydrides and lightweight complex metal hydrides hold significant potential for practical energy storage due to their high hydrogen capacity. However, one major drawback is their sluggish kinetics.^[^ ^1^ ^]^ Among these materials, magnesium hydride MgH_2_ stands out with its substantial gravimetric hydrogen storage capacity of 7.6 wt.% and extensive empirical database on structure and kinetic properties.^[^ ^2^ ^]^ Consequently, the Mg–H system serves as a model system for understanding key processes in hydrogen storage based on p‐metal hydrides. It exhibits four different stable phases: gas, liquid, solid solution α‐Mg, and the ionic hydride β‐MgH_2_, in contrast to metallic d‐metal hydrides.^[^ ^3^ ^]^ The direct transformation from metallic Mg to the stable rutile MgH_2_ involves a significant volume expansion of 32%.^[^ ^4^, ^5^ ^]^ In addition to its relevance in hydrogen storage, this transition also presents an opportunity for studying switchable optical, plasmonic, and nanophotonic systems due to the drastic change in optical properties.^[^ ^6^, ^7^ ^]^ Despite the potential of the material, their practical use is hindered by slow hydrogen sorption kinetics. One reason for these slow kinetics is the large activation barrier for hydrogen dissociation on Mg.^[^ ^8^, ^9^, ^10^, ^11^, ^12^, ^13^ ^]^ This issue can be viewed similarly to hydrogen spillover to metal oxides, such as WO_3_, that only absorbs hydrogen in presence of a noble metal catalyst.^[^ ^14^, ^15^, ^16^ ^]^ Significant efforts have been invested in finding such catalysts that improve molecular hydrogen dissociation and atomic hydrogen diffusion.^[^ ^17^, ^18^ ^]^ This comprises both the scientific background^[^ ^19^, ^20^ ^]^ as well as the machinery.^[^ ^8^, ^21^, ^22^ ^]^ However, the behavior of certain empirically discovered active additives challenges the established concepts of lowering H_2_ dissociation barriers and improving H diffusion, leading to debates on their classification as catalysts.^[^ ^23^ ^]^ Striking examples are Ti additives accelerating hydrogen sorption in NaAlH_4_,^[^ ^24^ ^]^ and oxide additives for hydrogen sorption in magnesium, where neither titanium nor transition metal oxides are typical hydrogenation catalysts.^[^ ^23^, ^25^ ^]^ Additionally, effective catalysts for many other potential hydrogen storage systems remain elusive.

### 1.1 Hydrogenation Behavior

The extremely slow hydrogen sorption kinetics in Mg are a severe constraint in basically all of its applications. An illustrative negative example is hydrogenation of Pd‐capped Mg thin films. A huge thermodynamic overpotential is required to completely transform a 180 nm thick Mg layer into MgH_2_^[^ ^26^ ^]^ (Δµ∝ln *p*_appl_ − ln *p*_plat_ with *p*_appl_ = 100 bar, *p*_plat_ ≃ 10^−3^ bar at *T* = 375 K), where *p*_appl_ is the applied hydrogen pressure and *p*_plat_ is the plateau pressure of the metal to hydride transformation). The dissociation of H_2_ at the surface of Mg is highly activated, ranging from 0.85 to 1.15 eV.^[^ ^8^, ^9^, ^10^, ^11^, ^12^, ^13^ ^]^ Surprisingly, overlayers or nanoparticles that reduce this barrier by catalyzing hydrogen dissociation, only marginally improve hydrogen sorption kinetics. This discrepancy is often attributed to the extremely slow diffusion of hydrogen in the hydride phase (*D* = 1.1 × 10^−20^m^2^s^‐1^ at T = 305 K).^[^ ^27^, ^28^, ^29^ ^]^ Hydrogen diffusion in the metal phase is relatively fast (*D* = 6.6 × 10^−9^m^2^s^‐1^ at 673 K^[^ ^30^, ^31^ ^]^); however, the low solubility of hydrogen in the α −phase reduces the overall permeation.^[^ ^29^, ^32^ ^]^ It is crucial to recognize that slow diffusion may merely be a symptom of the sluggish phase transformation necessitating a significant overpotential.

This complexity gives rise to various hydrogenation behaviors, such as the development of a blocking layer, leaving room for multiple interpretations of the kinetics based on hydrogen transport across different phases and structures.^[^ ^29^, ^33^, ^34^ ^]^ To address the challenges associated with sluggish kinetics, numerous kinetic models have been proposed, incorporating hydrogen dissociation, chemisorption, surface–subsurface penetration, bulk diffusion, and phase transformation a a rate limiting step. The improvement of individual reaction steps, such as employing metal catalysts for hydrogen dissociation or enhancing diffusion through dopants, holds promise for kinetic enhancement.^[^ ^25^, ^35^, ^36^, ^37^ ^]^ However, significant progress has been primarily achieved at high temperatures. Here, we present a completely new perspective on hydrogen sorption in ionic hydrides focusing on the archetypal example MgH_2_, which will help in the development of new catalysts. For this, we study the electronic structure of the MgH_2_ system derived from DFT calculations with a particular focus on the charge distribution in partially hydrogenated systems. The results reveal that the hypothetical MgH_0 < *x* < 2_ surfaces form ionic MgH_2_ alike hydrogen rich surface phases in contact with hydrogen free Mg bulk or surfaces. This corresponds to the experimentally observed behavior of bulk Mg–MgH_2_, which is an almost binary phase diagram of very low hydrogen solubility in the metallic phase, and a low hydrogen vacancy concentration in the hydride phase. Crucially, these surface phenomena significantly influence the kinetics of hydrogen sorption in Mg. Local hydrogen dissociation, as enabled by, e.g., noble metal nanoparticles, will result in small unstable MgH_2_ islands, necessitating a substantial oversaturation to compensate the endothermicity before the stable complete MgH_2_ surface is formed. Kinetic studies indicating the so‐called shrinking envelope kinetics corroborate these theoretical results.^[^ ^23^, ^25^, ^29^ ^]^ Experimental validation of such surface phenomena is challenging. To provide deeper insights into the surface phenomena during hydride growth, we designed an experimental setup utilizing reflecting electron energy loss spectroscopy (REELS) for surface characterization of the Mg hydrogenation process under oxygen free operating conditions. By probing surface plasmons, which exhibit extreme surface sensitivity, our measurements confirm the anticipated segregation behavior between hydrogen‐free metallic Mg and hydrogen‐covered insulating MgH_*x*_ surfaces. This explains the failure of classical hydrogenation catalysts as only the vicinity of the catalyst is readily hydrogenated; with the induced electrical polarization between hydride and metallic Mg acting as a barrier for further hydrogenation.

## 2 Results and Discussion

### 2.1 Charge Transfer at the Mg–MgH_2_ Interface by DFT Calculations

**Figure 1:**
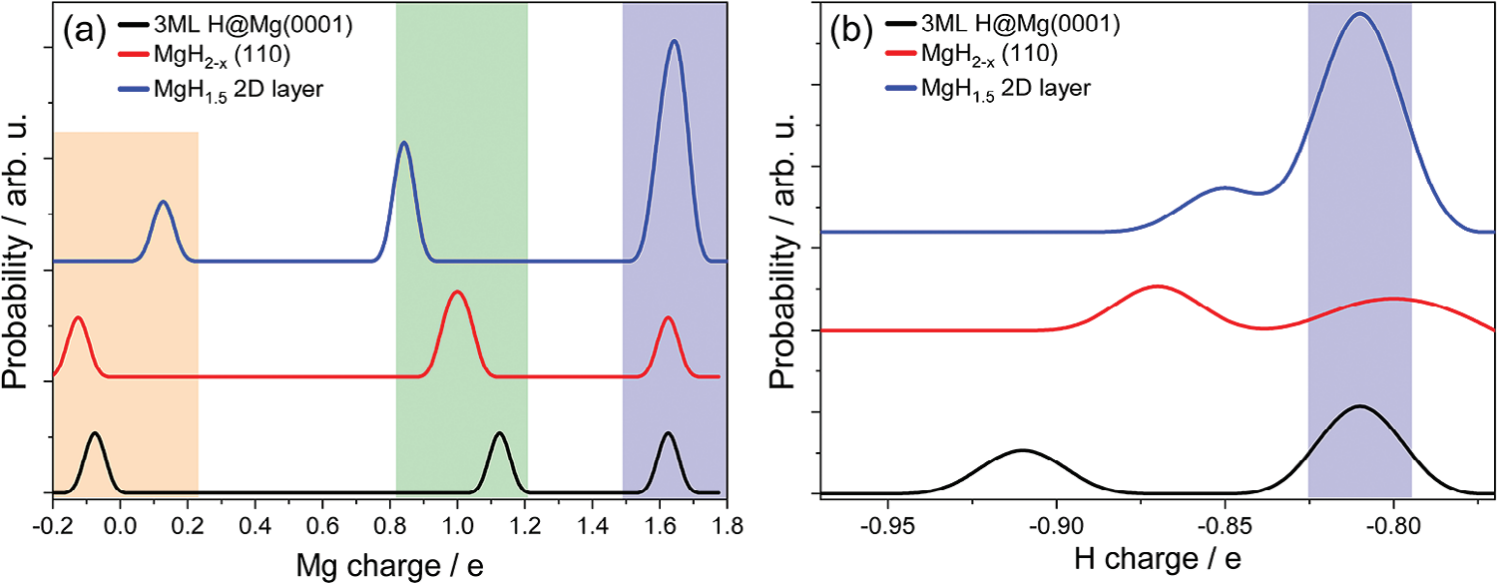
The calculated Bader charges on Mg (a) and H (b). Black represents 3 ML of hydrogen adsorbed on Mg(0001) surface; red corresponds to H deficient MgH_2_(110) surface, where 50% H vacancies are present at the surface; blue indicates a model system of the single MgH_1.5_ layer. The shaded orange, green, and blue regions refer to formal oxidation states of Mg 0, +1, and +2, respectively or ‐1 for H (b). The lines are drawn with broadening of 0.2e for Mg and 0.02e for H. For details of the charge distribution on atoms refer to Supporting Information and Figure S2, S3, S13, and S14 (Supporti ng Informationd).

In order to gain insight into magnesium hydride formation, we study three different model systems and discuss the charge distributions in various substoichiometric MgH_*x*_ compositions. These models include: i) hydrogen adsorption on Mg surfaces; ii) on the Mg (0001) isolated layer as well as iii) desorption of hydrogen from MgH_2_ (110). All of which are established model systems for DFT calculations on MgH_2_.^[^ ^3^, ^10^, ^20^, ^38^ ^]^ It is shown that hydrogen adsorption on Mg(0001) leads to the formation of weakly bound MgH_2_ layers. This allows to study model (ii) that is (4 × 4*R*45) supercell with a surface area of 2.82 nm^2^ and the single MgH_2_ layer. This single layer model allows focusing on the electronic effects that are related to Mg/H interaction, rather than on strain induced by hydrogen absorption. Furthermore it reveals that MgH_2_ clusters are significantly more stable than individual hydrogen atoms within the magnesium surface. Experimental charges of ionic MgH_2_ were measured by synchrotron X‐ray diffraction (XRD) and determined as +1.91 *e* for Mg and −0.26 *e* for H.^[^ ^39^ ^]^ The authors explained the apparent violation of charge neutrality by weak covalent bonding.^[^ ^40^ ^]^ Atomic‐scale calculations report charges from +1.95 *e* to +1.6 *e* for Mg and from −0.943 *e* to −0.6 *e* for H.^[^ ^41^, ^42^, ^43^ ^]^ The present calculations reveal the charges Mg^+1.62^ and H^−0.81^ (see also Table S3, Supporting Information) in bulk MgH_2_. The obvious difference to the experimental values given above may come from the experimental uncertainty of attributing the specific volume to Mg and hydrogen, respectively.^[^ ^39^ ^]^ As the probing X‐rays are scattered by all electrons, the resulting valence charges obtained from differences between neutral atoms and ions are error prone. The theoretical values are obtained with the Bader method.^[^ ^44^, ^45^ ^]^ The Bader charge are less prone to errors, because the boundary between the atoms is given by the surface of charge minima. While there are other ways of the electron distribution characterization in the ionic systems the charge distribution analysis applied here consistently compares changes of the ionic charges due to varying MgH_*x*_ stoichiometry in all model systems. Analysis of the charge distribution reveals the following: adsorption of hydrogen at Mg(0001) surface, formation of hydrogen vacancies on MgH_2_ (110) surface or formation of MgH_1.5_ atomic layer results in three distinguished magnesium charge states in the vicinity of H. They correspond to the formal charges 0, +1, +2, see **Figure** **1**a.

Multiple oxidation states are uncommon for p‐metals, unlike in transition metals. Mg^+1^ is known in molecular complexes with a particular ligand coordination and in presence of a strong Lewis base.^[^ ^46^ ^]^ The H^−^ anion is such a Lewis base thus low oxidation states of Mg can be anticipated. The charge distributions presented in Figure 1 reveal that the charge of magnesium atoms can range from 0 (metal) to +1.62 *e* (hydride) with additional states for non‐stoichiometric compositions. According to the present calculations the hydrogen atoms in contact with Mg are close to −1 irrespective their position in the system or the system's stoichiometry. At large deviations from stoichiometric MgH_2_ or sub‐monolayer H adsorption on Mg surface an electron transfer to hydrogen appears larger than 1 *e* for isolated hydrogen atoms (Figure 1b). Not dissociated H_2_ molecules at the surface remain neutral, for more details of the charge distribution for the intermediate H coverages we refer to the Supporting Information. Previous studies of hydrogen adsorption on Mg(0001)/($10\bar{1}3$) resulted in adsorption energies from ‐0.05 eV H^‐1^ at 1/4 H monolayer (ML) to ‐0.20 eV H^‐1^ for the full ML.^[^ ^38^, ^43^ ^]^ It was suggested that adsorption of > 5 ML of hydrogen on Mg(0001) leads to metal transformation to MgH_2_.^[^ ^38^ ^]^ This agrees with the present calculations that focus on adsorption of larger amounts of H.

**Figure 2:**
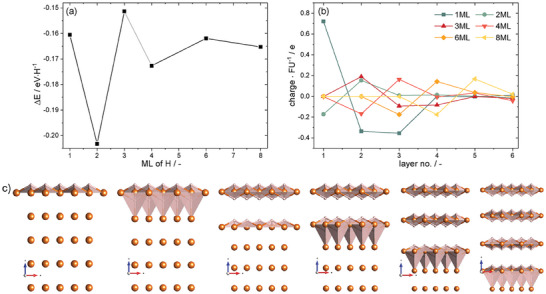
The adsorption energies for H on Mg(0001) surface, ML stands for full monolayer adsorption (a). Even numbers of layers indicate MgH_2_ double layer that is weakly bounded with Mg. The ionic charges averaged per formula unit of each separate layer (b). The layers are counted from the top and layer no. refers to Mg; MgH (for 1ML and 3ML); and MgH_2_ for those cases where they exist. The bottom panel (c) shows visualizations of the adsorption geometries for 1, 2, 3, 4, 6, and 8ML (left to right) where increasing separation between layers is self‐evident.

Adsorption of 2 ML hydrogen (or any even number of ML) on Mg(0001) results in MgH_2_ like structures that are more stable than 1 ML (or any odd number of ML), **Figure** **2**a. The surface layer with 2 ML H adsorbed has the electronic structure very similar to the bulk hydride and the charge distribution between anions and cations is identical in both systems. Mg^+1^ ions are present at the surface only for an odd number of H layers, and the topmost Mg‐H layer is positively charged, Figure 2b. This induces electron transfer to the underlying Mg. For even numbers of H ML adsorbed on Mg(0001), a small charge transfer is observed from Mg to adjacent MgH_2_ layer that is related to the differences of the work functions for these two materials. Moreover, the adsorption of consecutive hydrogen layers results in weakly bound MgH_2_ planes; the increasing layer separation manifests itself in the lattice expansion upon hydrogenation (Figure 2c and Supporting Information).

**Figure 3:**
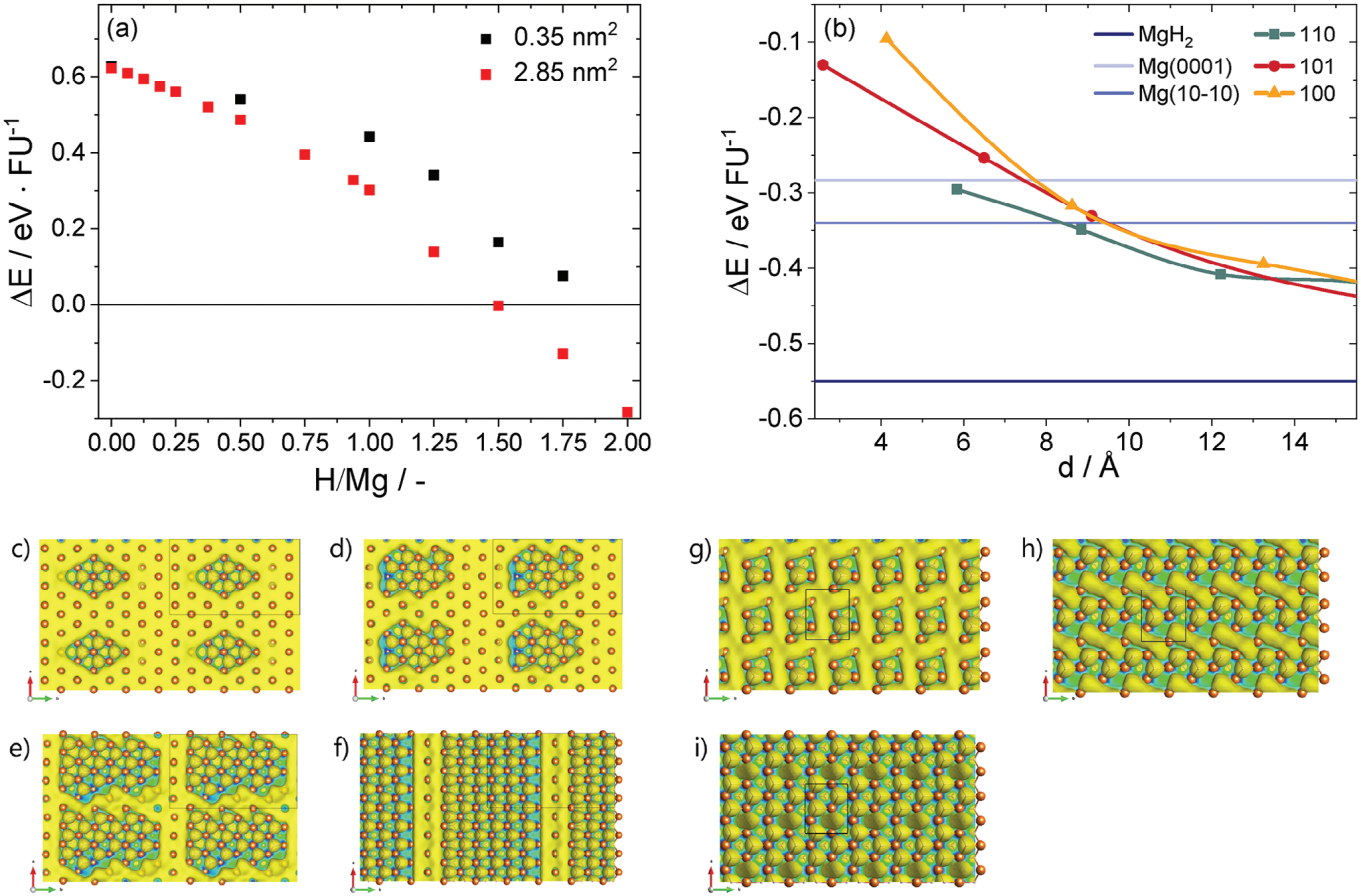
a) The stoichiometry dependent formation energies of MgH_*x*_ (0 ⩽ *x* ⩽ 2) on Mg(0001) single surface layer calculated using a large supercell (red). Black symbols represent the same calculations with a small supercell that does not allow for cluster formation. Panel b) depicts thickness dependence of the formation energy of MgH_2_ slabs of (110), (101), and (100) terminations compared to the energy of hydrogenated Mg(0001), Mg($10\bar{1}0$) or bulk MgH_2_ (horizontal lines). The hydrogen adsorption geometries are visualized by the electron localization function at 0.55. Large supercells with x=0.25 (c), 0.5 (d,g), 1.0 (e,h), and 1.5 (f,i) exhibit cluster formation (green) and presence of extended metallic magnesium (yellow). In smaller supercells with *x* ⩾ 0.5 (g‐i), metallic magnesium vanishes at larger hydrogen concentrations since MgH_2_ cluster cannot be formed.

As there is a thermodynamic preference for MgH_2_ like layers formation further insight is provided with the model single layer MgH_*x*_ on Mg(0001) defined above as (ii). This model has a large lateral dimension, thus it allows distinction between MgH_*x*_ cluster or uniform H adsorption. The formation energies of such MgH_*x*_ systems within the Mg layer are presented in **Figure** **3** where three observations can be made. First, only systems with hydrogen content larger than MgH_1.5_ are thermodynamically stable; second for any intermediate stoichiometry MgH_*x*_ clusters are more stable than uniformly distributed H, the preference can reach up to 0.4 eV per hydrogen atom (see also Supporting Information); third comparison of the stability of such MgH_2_ with MgH_2_(110), MgH_2_(101), and MgH_2_(100) indicate that for thickness larger than 5.5 Å  MgH_2_ becomes thermodynamically stable. This means that the slab exposing with given surface at such thickness is more stable than a sequence of MgH_2_ layers on Mg(0001), as shown in Figure 2c. For Mg($10\bar{1}0$) facet the maximum thickness of adsorbed H is 8 Å, for more details see Supporting Information. Within this considerations the lattice strains are relaxed at no cost, see Figure 2. In the bulk Mg such elastic relaxation is not possible and the direct insertion of hydrogen into Mg hexagonal lattice is an endothermic process. The H interstitial at tetrahedral void has energy penalty Δ*E* = 0.23 eVH^‐1^ (0.31 eV H^‐1^ at the octahedral one). This energy penalty decreases threefold until elastic energy dominates destabilizing H clusters (Figure S8, Supporting Information). Thus, a simple formation of MgH_2_ within Mg bulk is thermodynamically unfavorable. Similar trend for MgH_2_ and Mg separation is found for dehydrogenation of MgH_2_(110). In such process H vacancy formation energy weakly depends on their density at the surface (up to 50%) but the strong energetic preference is observed for formation of metallic Mg/MgH_2_ interface rather than uniform vacancy distribution on the surface, see Supporting Information.

In summary, the presented calculations provide evidence that partial hydrogenation of magnesium leads to the formation of localized MgH_2_ clusters. Such clusters, in contact with metallic Mg, are thermodynamically more stable than sub‐stoichiometric hydride with isolated hydrogen atoms. These hydride clusters induce Mg^+1^ and other fractional charges at the interface. Overall, slightly hydrogen deficient clusters carry positive charge that is compensated by an excess of electrons in adjacent Mg. This interface polarization may inhibit the mobility of H^−^ ions and explain in part the large thermodynamic overpotential necessary to induce the Mg–MgH_2_ phase transition.

Moreover, the positive formation energy of the H interstitial within Mg effectively restricts direct formation of the hydride by diffusion of hydrogen into Mg. The strong tendency of hydrogen clustering in the bulk magnesium combined with elastic strain effects calls for the revision of H diffusion in Mg. This might be a collective phenomenon, where diffusion of isolated hydrogen atoms accounts for small cluster formation only. However, expansion of the clusters is related to the lattice strains and Mg– H cluster diffusion could be a more complex phenomenon than single atom diffusion. In the discussion below we address the connection between activation energy and the charge transfer.

### 2.2 Experimental Determination of Surface Hydrogen

**Figure 4:**
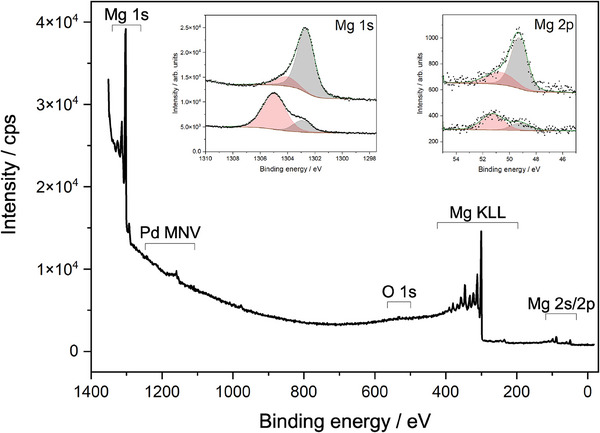
Mg XPS Survey and details of Mg 1s and 2s peaks of the thin film as grown (black) and after hydrogenation (red), respectively, confirming that the film was not oxidized. The 2.3 eV shift of the Mg 1s peak (detail in left inset) upon hydrogenation is consistent with expectations from the literature.^[^ ^27^ ^]^

Experimental verification of the above mentioned ideas requires an operando surface science approach. X‐ray photoelectron spectroscopy is the usual tool for this (see, e.g., Refs. [12, 27, 47]). However, hydrogen has no core‐level electrons,^[^ ^16^ ^]^ and thus the detection can only be measured indirectly by changes of the Mg core‐electrons (chemical shift of Mg 1s and 2p, see insets in **Figure** **4**).^[^ ^27^ ^]^ Hydrogen chemisorbed on metallic Mg can thus hardly be distinguished from (bulk) MgH_2_, because a significant shift occurs upon strong interaction, as is the case for ionic MgH_2_ alone. Furthermore, although considered to be surface sensitive, the information of XPS is several monolayers, impeding clear distinction between surface and subsurface changes. To overcome this barrier, we apply reflecting electron energy loss spectroscopy (REELS) on Mg under hydrogenation conditions. Similar to XPS, the energy and intensity changes of plasmons probed by REELS provide surface sensitive chemical information. In addition, the peculiarity of quasi‐free electron systems such as Mg is the occurrence of strong surface/interface plasmons, which are very sensitive to electronic structure.

Thin Mg films (≈ 30 nm) were grown on Pd membranes by magnetron sputtering (see Section 4). The films are hydrogenated with atomic hydrogen through application of a high hydrogen pressure on the feed side (*p*_feed_) of the Pd membrane.^[^ ^48^ ^]^ Hydrogen diffuses through the membrane as atomic hydrogen, and further to the Mg layer on top of it. Due to the slow permeation kinetics, the pressure in the vacuum vessel remains compatible to typical surface science methods. The high quality of the films, in particular the very low oxygen content is confirmed by in situ XPS measurements (Figure 4). Hydrogenation leads to the expected shift of the Mg 1s peak in good agreement with results gained on a similar setup.^[^ ^27^ ^]^ The evolution of the chemical shift is in agreement with the discrete formation of MgH_2_ co‐existing with Mg. However, further statements on surface species on Mg cannot be drawn.

REELS is an established technique to probe the electronic structure of the extended surface of solids, i.e., the topmost surface layer up to a depth of a few nanometers.^[^ ^49^ ^]^ Electrons with a defined primary energy *E*_*i*_ are reflected by such an extended surface, and their number and energy loss detected by an electron analyzer. During the scattering process, energy can be transferred to electrons (interband and core‐electron transition), atoms (so‐called recoil process), and quasi‐particles (plasmons and phonons) resulting in peaks in the loss spectrum. At the given primary energy and electron monochromator/spectrometer resolution, scattering by plasmons is the dominant process. The fundamental energy of a plasmon ω_p_ (eigen frequency) is given by

(1) $$\omega_{p}^{2}=\frac{ne^{2}}{m\tilde{\varepsilon_{0}}}$$

with the number of free electrons *n*, the mass of the electron *m*, and the permittivity of the material $\tilde{\varepsilon_{0}}$. As long as ω < ω_p_, coherent movements of electrons are strongly damped, since the electrons are screened against each other by the compensating positive polarization cloud around them. In the case where ω ⩾ ω_p_, the electrons feel the long‐range Coulomb interaction, and undamped plasma oscillations occur.^[^ ^50^ ^]^ In undamped systems, *n*_o_ overtones of the eigenfrequency ω_p_ occur.

Electrons in metallic magnesium are well described by the free electrons as observed by the strong undamped plasma oscillations ω_b_ = *n*_o_ × 11 eV with 1< *n*_o_ < 5 (see Figure S1 Supporting Information). Upon hydrogenation, a new peak appears at ℏω ≈ 14.5 eV, which is the bulk plasmon of MgH_2_.^[^ ^52^, ^53^, ^54^, ^55^ ^]^ In ionic MgH_2_, only bound electrons exist, and the free electron model is no longer viable. Nonetheless, plasmonic oscillations are observed in MgH_2_, due to bound electrons undergoing interband transitions and thus becoming “free”.^[^ ^54^ ^]^ The fundamental plasmon peaks observed by low energy REELS (Figure S1, Supporting Information) agree very well with high energy EELS as used with transmission electron microscopy (TEM).

In addition to the excitation of bulk plasmons, surface plasmons can be excited. The surface plasmon is essentially a 2D wave in contrast to the 3D volume plasmon, and thus, the energy of the surface excitation in the free electron model is expected at^[^ ^56^, ^57^ ^]^

(2) $$\omega_{s}=\omega_{b}/{\left(1+\varepsilon_{r}\right)}^{1/2}=\omega_{b}\cdot k/\sqrt{2}$$

with ϵ_*r*_ the relative dielectric constant of the boundary layer. In case of an unknown interface, we introduce the value *k* as the parameter describing its deviation from an ideal metal– vacuum interface. For pristine Mg it is *k* = 0.960 (see Figure 6), confirming the clean metallic Mg– vacuum interface.

**Figure 5:**
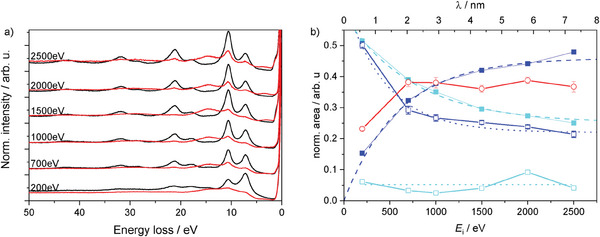
a) REELS of Mg before (black) and after hydrogenation (red) for different incident electron energies *E*_*i*_ = 200– 2500 eV recorded at *T* = 85 °C and with *p*_feed_ = 10 mbar. In the metallic state, there is a clear incident energy dependence of $I_{\omega_{b}}/I_{\omega_{s}}$. Furthermore, the elastic recoil peak of hydrogen (≈ 4eV) increases in intensity with increasing *E*_*i*_ and shifts toward higher energy. b) Normalized area of Mg bulk (blue), surface (turquoise), and hydride (red) plasmons before (filled symbols) and after hydrogenation (empty symbols) for different incident electron energies. The top x‐axis denotes the inelastic mean free path of electrons through Mg at the corresponding energies.^[^ ^51^ ^]^ Dashed (before hydrogenation) and dotted lines (after hydrogenation) are a guide to the eye. Error bars indicate one standard deviation derived from Monte Carlo simulations for the fits within CasaXPS.

**Figure 6:**
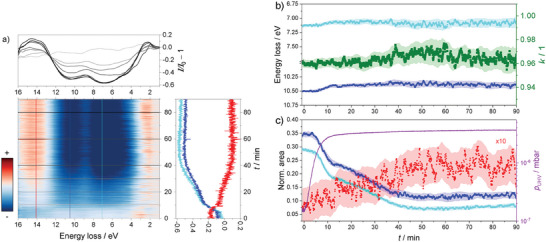
a) Time‐resolved REELS difference spectra recorded at *E*_*i*_ = 1000 eV during hydrogen absorption at *T* = 85°C and with *p*_f_ = 10 mbar shown as a heatmap. Horizontal line scans highlight the corresponding spectra. The vertical line scans through the 2D plot reveal the intensity loss and gain of the Mg‐surface and bulk, and MgH_2_ plasmons in blue, turquoise, and red, respectively. b) Time evolution of REELS peak areas for bulk (dark blue), surface (turquoise), and hydride (red) plasmons during hydrogenation, extracted from spectra in Figure 6a. The pressure in the analysis chamber is represented in purple. c) Time evolution of REELS peak positions for magnesium bulk (dark blue) and surface (turquoise) plasmons. The correction factor *k* (Equation (3)) is depicted in green. Shaded areas indicate one standard deviation.

With energies below 3 keV, REELS probes the surface down to a depth of around 3 nm. In contrast, the surface plasmons are confined to the surface; in particular, the surface sensitivity is higher than the mean free path length of the electrons.^[^ ^49^ ^]^ Small fractions of a monolayer coverage are sufficient to change the surface plasmon.^[^ ^58^, ^59^, ^60^, ^61^ ^]^ By varying the incident energy of the electrons, the information depth of the bulk plasmon‐excitation varies, while the intensity of the surface plasmons is only affected by the energy‐dependent scattering process.^[^ ^49^ ^]^ We use this to probe the surface structure before and after hydrogenation. In pure Mg, the intensity of the surface plasmon decreases with increasing incident electron energy (**Figure** **5**). After hydrogenation, a small amount of metallic Mg remains as evidenced by the remaining Mg plasmons. Their energy dependence indicates that this Mg is a thin Mg layer on top of a bulk MgH_2_ layer: the MgH_2_ plasmon slightly increases with energy with the Mg bulk plasmon decreasing (Figure 5b). The intensity of the Mg‐surface plasmon remains nearly constant, what is in line with the model that the excitation stems from the surface above the Mg layer and the interface between it. The model is in agreement with the observed temporal evolution of REELS spectra during hydrogenation (**Figure** **6**).

Although anticipated by the calculations (Section 2.1), the formation of a metallic Mg layer on top of a hydride is somewhat surprising. At the partial pressures used (*p* ⩾ 10^−5^ mbar), typical hydride forming d‐metals (Pd, Ti, etc.) chemisorb hydrogen very strongly.^[^ ^19^ ^]^ To corroborate the findings by theory, we have to prove that the top Mg layer has no hydrogen bound to it. Chemisorbed hydrogen can only be detected indirectly by electron spectroscopy. The surface plasmons are very sensitive to any perturbation of the surface, be it roughness or any change of the electronic structure by, e.g., chemisorbed atoms. Changes of the surface plasmon can be used to quantify hydrogen on the surface, if other adsorbates (such as oxygen and carbon) can be excluded by XPS. The general idea was first experimentally shown by Voskoboinikov et al., who used the intensity ratio between surface and bulk plasmon to quantify the oxygen coverage.^[^ ^58^ ^]^ However, intensity calculation as well as experimental uncertainties impede an absolute quantification. Therefore, Alducin et al.^[^ ^60^ ^]^ and later Li et al.^[^ ^59^ ^]^ made use of the energy dependence of the surface plasmon on oxygen coverage to independently quantify the oxygen coverage on aluminum. For this, they specified Equation (3) using the frequency‐dependent polarizability model developed by Feibelman,^[^ ^61^ ^]^ which is valid for the submonolayer regime:

(3) $$\omega_{s}=\frac{\omega_{b}}{\sqrt{2}}\underset{=k}{\underset{︸}{\left(1-\frac{\beta d_{⊥}}{2}\right)}}$$

β is the invariant propagation constant, *d*_⊥_ is the centroid of the induced charge calculated from the dispersion relation of surface plasmon in the quasi‐static limit.^[^ ^60^, ^61^ ^]^

The model was verified for oxygen on Al surfaces by the independent determination of the oxygen coverage using XPS.^[^ ^59^, ^60^ ^]^ This procedure is not possible for hydrogen on Mg. We thus rely on the assumption that β and *d*_⊥_ are similar for both systems, which is likely as Al_2_O_3_, MgO, and MgH_2_ have similar electronic structures (e.g., bandgap,^[^ ^5^, ^54^ ^]^ see also projected DOS in Figure S12, Supporting Information). We can make use of the experimental observation of Alducin et al.^[^ ^60^ ^]^ and Li et al.,^[^ ^59^ ^]^ that already sub‐monolayer coverage leads to shifts of the surface plasmon frequency in the eV range, which is easily measurable. We thus discuss the results along the above introduced factor *k*: any deviation from unity hints toward a change of the interface, such as adsorption of hydrogen.

Figure 6 shows a typical evolution of REELS spectra during the hydrogenation of Mg as 2D plot. Horizontal scans are the measured spectra, vertical scans reveal the change of intensities indicative for Mg bulk and surface plasmons and the MgH_2_. Fitting of the three peaks by CasaXPS reveals MgH_2_ overall concentration (Figure 6), slowly growing over time. The main aspect of this study is revealed by the ratio of the Mg surface to bulk frequencies (k = $\omega_{s}\cdot \sqrt{2}/(\omega_{b}$)) (Equation (3)). The quality factor *k* ≃ 1 remains constant over the course of the reaction despite an increase in MgH_2_ (and thus decrease of Mg), and a decrease of the ratio of the Mg surface to bulk intensity. This allows a first qualitative statement: hydrogenation including the surface and bulk includes only two states: a clean Mg and a MgH_2_ state.

**Figure 7:**
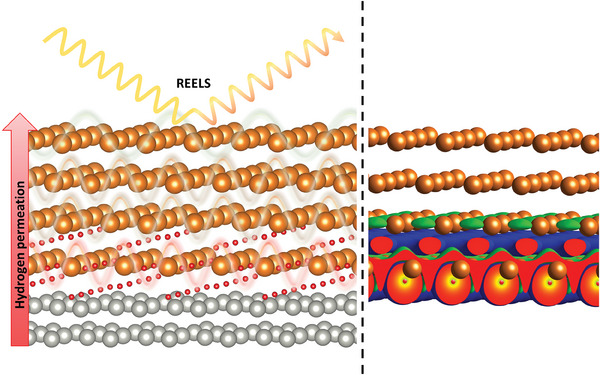
Illustration of principal idea and main outcome. Left: Simplified experimental scheme highlighting the hydrogenation of the Mg layer through the supporting Pd membrane and the main spectroscopic method used to probe plasmons (waves) being sensitive to the hydrogen in/on the layer. Right: Charge density difference images at an MgH_2_–Mg interface by DFT calculations. Hydrogen in MgH_2_ is negatively charged, Mg is positive. At the interface, the ionic compounds polarize the adjacent metallic Mg layer.

A negligible hydrogen coverage on Mg as deduced from the constant quality factor at pressures of *p* > 10^−6^ mbar is in perfect agreement with the theory results: substoichiometric hydrogen coverage on Mg is unstable (Figure 3). The Mg layers sit on top of a MgH_2_ film (see **Figure** **7** for illustration). Despite its small thickness (around 1.2 nm as estimated from an exponential fit to the REELS intensity ratio in Figure 5), the electronic structure in the Mg‐layer is unaffected by the adjacent MgH_2_ (Figure 6). This may be surprising when comparing these results with metal/metal hydride interfaces such as Mo/VH_*x*_ and Fe/V_*x*_ layers.^[^ ^62^ ^]^ In these systems, hydrogen 1s electrons bind locally to the d‐electrons of the transition metal.^[^ ^63^ ^]^ Here, however, the electronic structure changes associated with hydrogen uptake in Mg are very different. As outlined in Section 2.1, hydrogen in Mg layers decomposes into hydrogen‐free Mg layers and MgH_2_ layers that are only weakly coupled (Figure 2). In particular, the charge density as the source of the plasmons in Mg is hardly affected, and thus a change in the REELS energies not expected as observed.

### 2.3 Hydrogen Sorption Kinetics in Mg

The experimental and theoretical results shed light on the stability of Mg– MgH_2_ interfaces at the nano‐scale. In many hydrogen– metal systems, the possibility of finite hydrogen solubility and finite vacancy density in the hydride phase enables the formation of a hydrogen gradient as the driving force of diffusion. The corresponding barrier is the microscopic hopping of hydrogen. Although such atomistic steps are relevant for diluted hydrogen diffusion in Mg and MgH_2_,^[^ ^27^, ^28^, ^29^, ^64^ ^]^ the instability of partially filled Mg layers (Figure 3) or small H clusters in Mg is a collective phenomenon adding another kinetic barrier. The existence of such an interface barrier has previously been deduced indirectly from modeling uptake kinetics.^[^ ^65^ ^]^ The latter is the dominant mechanism at small hydride thickness before diffusion through the MgH_2_ layer becomes rate‐limiting.^[^ ^65^ ^]^ In order to gain basic insight into kinetics of hydrogenation we have calculated the activation energies *E*_*a*_ for diffusion of H atoms through our model system that include hydrogen passing through Mg(0001) layer (Figures S10– S14, Supporting Information), H‐vacancy and H_2_ molecule hoping though MgH_2_ layer (Figures S12 and S15). Interestingly *E*_*a*_ is of the range of 0.4 eV for Mg(0001) and MgH_2_. These low barriers are related to small changes of the ionic charge on nearest Mg and negligible influence of the lattice strains. On the contrary hydrogen hoping at the Mg/MgH_2_ interface is related to a significantly larger activation energy *E*_*a*_ = 0.88 ‐1.30 eV once Mg changes from metallic to Mg^+1^ or Mg^+1^ to Mg^+2^/Mg, respectively (Figures S13 and S14, Supporting Information). The barrier of ≈1.4 eV for H_2_ dissociation/hoping on MgH_2_ is related large charge transfers toward H, see Supporting Information.

The existence of a metallic Mg layer on top of MgH_2_ generated during dynamic equilibrium is worth mentioning. The Mg layer is maximally exposed to UHV‐compatible hydrogen pressures (*p* < 10^−5^ mbar), which are below the plateau pressure. The growth of a MgH_2_ layer is still possible under dynamic conditions as applied in Figure 6, because the local chemical potential in the sample is higher than required to form the hydride.^[^ ^66^ ^]^ The local chemical potential is defined by chemical potential at the membrane feed side (which is well above the plateau pressure), and the flux of hydrogen through the entire thin film system, which depends on the kinetics. If the kinetics was desorption limited, the complete Mg film would be hydrogenated; in particular the top layer would be MgH_2_, too.^[^ ^48^ ^]^ As this is not the case, we can conclude that the kinetic constraint of hydrogen recombination at the Mg surface is lower than that of hydrogen diffusion through MgH_2_ and the Mg– MgH_2_ interface reaction. Despite the high dissociation barrier of dihydrogen on Mg,^[^ ^10^ ^]^ the impact of hydrogen dissociation catalysts on hydrogen sorption kinetics is thus limited.

How can we improve the sluggish hydrogen kinetics? The theoretical basis provides some input. Figures 7 and 3 pinpoint that the charge transfer of electrons from Mg to hydrogen is the main mechanism describing the stability of the formed layers and the interaction between Mg and MgH_2_. It is also major contributor to the activation energy for H hoping through Mg/MgH_2_ interface. As the interface moves with growth, a potential “catalyst” should be better described as “dopant”, either moving with the interface, or equally distributed within the entire sample. Dopants had been suggested to improve hydrogen diffusion in MgH_2_ by Hao et al.^[^ ^67^ ^]^ However, the experimental implementation did not show a clear effect.^[^ ^68^ ^]^ This may be due to the difficulty of avoiding segregation of distributed dopants such as Cu in Mg, and due to the fact that the interface reaction rather than diffusion is the rate‐limiting step^[^ ^65^ ^]^ in technical MgH_2_ storage materials due to their small grain size.^[^ ^25^ ^]^ Dopants affecting the Mg– MgH_2_ interface can be drawn from the theoretical modelling (Figure 7), however, the practical implementation remains yet unsolved.

In simple terms, we have shown that the origin of the sluggish kinetics lies in a drastic change of the electronic structure of Mg as it transforms into MgH_2_. This change is fundamental in a variety of other hydrogen metals, such as hydrogen‐alkaline and alkaline earth metal systems and complex hydrides. Both experimental methods and theory are therefore applicable to these systems.

## 3 Conclusion

Using a combined approach of DFT calculations and a dedicated experimental setup, we have studied the Mg– MgH_2_ interface during hydrogen sorption in Mg. To circumvent the challenge of high pressure to overcome hydrogen dissociation and the thermodynamic potential in conflict with chemical surface analysis by electron spectroscopy, Mg thin films deposited on Pd membranes are hydrogenated by atomic delivery of hydrogen through the membrane. Time‐resolved REELS measurements under oxygen‐free conditions provide an atomistic picture of the Mg– MgH_2_ interface evolution during hydrogen uptake, since the technique employed allows simultaneous measurement of bulk and surface. In particular, we find experimental evidence that the surface remains partially free of adsorbed hydrogen (thus excluding hydrogenation from the gas phase) and that the separation into Mg and MgH_2_ slows down the sorption kinetics even when atomic hydrogen is provided by surface catalysts.

DFT calculations provide a deeper understanding of the experimental results: partial hydrogenation of magnesium leads to the formation of localized MgH_2_ clusters. These clusters induce Mg^+1^ states at the interface, which in turn induce electron transfer toward metallic magnesium. The clusters effectively carry a positive charge, which is compensated for by an excess of electrons in neighboring Mg. This interfacial polarization can inhibit the mobility of H^−^ ions and partly explains the large thermodynamic overpotential required to induce the Mg– MgH_2_ phase transition. In addition, the positive formation energy of interstitial H in Mg effectively limits direct formation of the hydride by diffusion of hydrogen into Mg.

Summarizing, the study sheds new light on sorption kinetics in MgH_2_ from an experimental, as well as theoretical point of view; in particular, it explains the limited effectiveness of classical hydrogenation catalysts, and points to the importance of the surface and bulk electronic structure of metallic Mg and insulating MgH_2_ for the underlying phase transformation.

## 4 Experimental Section

### UHV Chamber

The permeation experiments as well as the operando surface analysis were performed in a tailored ultra‐high vacuum (UHV) chamber with a preparation and an analysis level with a base pressure of around 10^−9^ mbar. Main chamber and electron analyzer were pumped by Pfeiffer HiPace 300 and HiPace 80 turbo pumps, respectively, to maintain UHV compatible pressures also under high hydrogen load introduced into the chamber by the membrane.

### Sample Preparation

The sample preparation level included a variable energy argon ion source (Leybold), a magnetron sputter deposition source (AJA A300 XP) and an active capacitance pressure gauge (Pfeiffer CMR 363). For sputter deposition/etching, argon was fed into the chamber continuously by mass flow controller. In order to achieve sufficiently high argon pressure in the chamber of around 10 Pa, the turbomolecular pumps were slowed down. Magnesium growth rate was around 1.6 nmmin^‐1^ at a total power of 40 W. Typical thickness of the investigated magnesium layers was 32 ± 4 nm. The magnesium sputter rate of 96 ± 12 nm h^−1^ was determined from XPS/HAXPES at different sputtering times (see Supporting Information). After each measurement cycle, the sputtered film was removed by Ar‐ion etching with 4 keV Ar ions. Each time, the purity of the cleaned palladium membrane, as well as of the newly deposited layer was checked by AES and XPS (Figure 4).

### Surface Analysis

The in situ analysis level consists of a VSW Class100 hemispherical electron analyzer equipped with a single channel electron multiplier. A dual anode X‐ray source (Prevac RS 40B1) was mounted at a 58° angle and a tuneable electron source (Specs EQ 22/35) was mounted at 50° with respect to the entrance of the electron analyzer. The spectrometer energy resolution (*E*_pass_ = 30 eV) was around 100 meV for XPS experiments. The energy resolution of reflecting electron energy loss spectroscopy (REELS) further degradates due to convolution with the primary electron beam energy width. Additionally, the chamber was equipped with a quadrupol mass analyzer (SRS RGA 100).

Ex situ XPS/HAXPES analysis was performed using a PHI Quantes spectrometer (ULVAC‐PHI), equipped with a conventional low‐energy Al‐Kα source (1486.6 eV) and a high energy Cr‐Kα (5414.7 eV) X‐ray source. Both sources were high flux focused monochromatic X‐ray beams that could be scanned across the sample surface to analyze a selected area on the sample surface. The energy scale of the hemispherical analyzer was calibrated according to ISO 15472 by referencing the Au 4f_7/2_ and Cu 2p_3/2_ main peaks (as measured in situ for corresponding sputter‐cleaned, high‐purity metal references) to the recommended BE positions of 83.96 and 932.62 eV, respectively. XPS survey spectra, covering a binding energy (BE) range from 0 to 1350 eV, were recorded with a step size of 1 eV at a constant pass energy of 280 eV using the Al‐Kα source (power 24.5 W; beam diameter 100 µ m). XPS detailed regions (i.e., Mg 1s, Mg 2s, O 1s, Si 2p) were extracted from the survey spectra. HAXPES survey spectra, covering a binding energy (BE) range from 0 to 5000 eV, were recorded with a step size of 1 eV at a constant pass energy of 280 eV using the Cr‐Kα source (51.6 W; beam diameter 91.6 µ m). HAXPES detailed regions (i.e., Mg 1s, Mg 2s, O 1s, Si 2p) were extracted from the survey spectra.

Inelastic mean free path (IMFP)‐calculations were performed using the QUASES‐IMFP‐TPP software^[^ ^51^ ^]^

### Data Processing and Statistical Analysis

The elastic recoil peak in the unprocessed REELS spectra was fitted in RStudio with a Gaussian peak shape, the center of which was shifted to 0 eV energy loss. All spectra were normalized to the peak area of the elastic recoil peak. The peak fitting analysis was performed in CasaXPS (version 2.3.24 PR1.0) and consists of Gaussian peaks fitted over a Shirley background from 1 to 35 eV energy loss. XPS/HAXPES spectra were analyzed using MultiPak (version 9.9) and CasaXPS. Mg 1s, Mg 2p, O 1s, Si 1s, and Si 2s regions were fitted using a Gaussian‐Lorentzian shape, with a Gaussian contribution of 70%. A standard Shirley background was used for all spectra. Peak models and uncertainties were assessed with the Monte Carlo routine for error estimation within CasaXPS. All uncertainties represent the mean ± standard deviation.

### Theoretical Calculations

The calculations were performed within Density Functional Theory (DFT) and the Vienna ab initio simulation package (VASP).^[^ ^69^, ^70^ ^]^ The atoms were represented with the projector augmented wave (PAW) potentials^[^ ^71^, ^72^ ^]^ with the valence configuration 2*p* ^6^3*s* ^2^ for Mg and 1*s* ^1^ for H. Exchange correlation functional was Perdew, Burke, Ernzerhof (PBE);^[^ ^73^ ^]^ the plane wave basis set energy cutoff was 500 eV, dense k‐point sampling of the reciprocal space was used. For the surface calculations the slab models with min. 15 Å  was used. Charge density analysis was done within Bader method,^[^ ^45^ ^]^ energy barriers were calculated with nudged elastic band (CI‐NEB) method.^[^ ^74^ ^]^ For additional details and accuracy assessment refer to Supporting Information.

## Author Contributions

S.K. and F.L. performed experimental work and data evaluation. E. B. performed conception of experiments and experimental work. A. B. performed conception of experiments and data evaluation. Z.Ł. performed conception of theory framework and DFT calculation. All authors contributed to writing of the manuscript.

## Conflict of Interest

The authors declare no conflict of interest.

## Supporting information

Supporting Information

## Data Availability

The data that support the findings of this study are openly available at https://doi.org/10.5281/zenodo.10276664, reference number 10276664.

## References

[1] L. Pasquini , K. Sakaki , E. Akiba , M. D. Allendorf , E. Alvares , J. R. Ares , D. Babai , M. Baricco , J. B. von Colbe , M. Bereznitsky , C. E. Buckley , Y. W. Cho , F. Cuevas , P. de Rango , E. M. Dematteis , R. V. Denys , M. Dornheim , J. F. Fernández , A. Hariyadi , B. C. Hauback , T. W. Heo , M. Hirscher , T. D. Humphries , J. Huot , I. Jacob , T. R. Jensen , P. Jerabek , S. Y. Kang , N. Keilbart , H. Kim , et al., Prog. Energy 2022, 4, 032007.

[2] M. Kappes , M. Iannuzzi , R. M. Carranza , J. Electrochem. Soc. 2013, 160, C168.

[3] S. Dong , C. Li , J. Wang , H. Liu , Z. Ding , Z. Gao , W. Yang , W. Lv , L. Wei , Y. Wu , H. Li , J. Mater. Chem. A 2022, 10, 22363.

[4] K. Zeng , T. Klassen , W. Oelerich , R. Bormann , Int. J. Hydrogen Energy 1999, 24, 989.

[5] J. Isidorsson , M. E. Giebels , R. Griessen , H. Arwin , Phys. Rev. B ‐ Condens. Matter Mater. Phys. 2003, 68, 1.

[6] P. Hjort , A. Krozer , B. Kasemo , J. Alloys Compd. 1996, 234, L11.

[7] J. Karst , F. Sterl , H. Linnenbank , T. Weiss , M. Hentschel , H. Giessen , Sci. Adv. 2020, 6, eaaz0566. 32494706 10.1126/sciadv.aaz0566 PMC7210000

[8] A. Borgschulte , M. Bielmann , A. Züttel , G. Barkhordarian , M. Dornheim , R. Bormann , Appl. Surf. Sci. 2008, 254, 2377.

[9] J. F. Stampfer , C. E. Holley , J. F. Suttle , J. Am. Chem. Soc. 1960, 82, 3504.

[10] T. Vegge , Phys. Rev. B 2004, 70, 035412.

[11] A. J. Du , S. C. Smith , X. D. Yao , G. Q. Lu , J. Phys. Chem. B 2005, 109, 18037. 16853316 10.1021/jp052804c

[12] M. Johansson , C. W. Ostenfeld , I. Chorkendorff , Phys. Rev. B 2006, 74, 193408.

[13] M. Pozzo , D. Alfè , J. Phys. Condensed Matter 2009, 21, 095004. 21817377 10.1088/0953-8984/21/9/095004

[14] A. R. Berzins , P. A. Sermon , Nature 1983, 303, 506.

[15] R. Prins , Chem. Rev. 2012, 112, 2714. 22324402 10.1021/cr200346z

[16] E. Billeter , A. Sterzi , O. Sambalova , R. Wick‐Joliat , C. Grazioli , M. Coreno , Y. Cheng , A. J. Ramirez‐Cuesta , A. Borgschulte , Phys. Rev. B 2021, 103, 205304.

[17] L. Schlapbach , R. Bowman , N. Gerard , H. K. Lotsch , Hydrogen in intermetallic compounds II: Surface and dynamic properties, applications, vol. 67, Springer, Berlin 1992.

[18] K.‐F. Aguey‐Zinsou , J.‐R. Ares‐Fernández , Energy Environ. Sci. 2010, 3, 526.

[19] K. Christmann , Surf. Sci. Rep. 1988, 9, 1.

[20] S. Kumar , T. Pavloudis , V. Singh , H. Nguyen , S. Steinhauer , C. Pursell , B. Clemens , J. Kioseoglou , P. Grammatikopoulos , M. Sowwan , Adv. Energy Mater. 2018, 8, 1.

[21] N. Hanada , T. Ichikawa , H. Fujii , J. Phys. Chem. B 2005, 109, 7188. 16851820 10.1021/jp044576c

[22] J. M. Bellosta von Colbe , W. Schmidt , M. Felderhoff , B. Bogdanović , F. Schüth , Angew. Chem., Int. Ed. 2006, 45, 3663. 10.1002/anie.200504425 16642519

[23] Q. Hou , X. Yang , J. Zhang , ChemistrySelect 2021, 6, 1589.

[24] T. J. Frankcombe , Chem. Rev. 2012, 112, 2164. 22166103 10.1021/cr2001838

[25] G. Barkhordarian , T. Klassen , R. Bormann , J. Alloys Compd. 2006, 407, 249.

[26] J. Isidorsson , M. E. Giebels , R. Griessen , H. Arwin , Phys. Rev. B Condens. Matter Mater. Phys. 2003, 68, 1.

[27] P. Spatz , H. A. Aebischer , A. Krozer , L. Schlapbach , Zeitschrift fur Physikalische Chemie 1993, 181, 393.

[28] J. Töpler , H. Buchner , H. Säufferer , K. Knorr , W. Prandl , J. Less‐Common Met. 1982, 88, 397.

[29] H. T. Uchida , S. Wagner , M. Hamm , J. Kürschner , R. Kirchheim , B. Hjörvarsson , A. Pundt , Acta Mater. 2015, 85, 279.

[30] A. Atrens , N. Winzer , G. Song , W. Dietzel , C. Blawert , Adv. Eng. Mater. 2006, 8, 749.

[31] H. Schimmel , G. Kearley , J. Huot , F. Mulder , J. Alloys Compounds 2005, 404‐406, 235.

[32] C. Nishimura , M. Komaki , M. Amano , J. Alloys Compd. 1999, 293‐295, 329.

[33] J. Rydén , B. Hjörvarsson , T. Ericsson , E. Karlsson , A. Krozer , B. Kasemo , J. Less‐Common Met. 1989, 152, 295.

[34] V. P. Zhdanov , A. Krozer , B. Kasemo , Phys. Rev. B 1993, 47, 11044. 10.1103/physrevb.47.11044 10005238

[35] P. S. Rudman , J. Appl. Phys. 1979, 50, 7195.

[36] M. H. Mintz , Y. Zeiri , J. Alloys Compd. 1995, 216, 159.

[37] T. Førde , J. Maehlen , V. Yartys , M. Lototsky , H. T. Uchida , Int. J. Hydrogen Energy 2007, 32, 1041.

[38] T. Jiang , L.‐X. Sun , W.‐X. Li , Phys. Rev. B 2010, 81, 035416.

[39] T. Noritake , S. Towata , M. Aoki , Y. Seno , Y. Hirose , E. Nishibori , M. Takata , M. Sakata , J. Alloys Compds. 2003, 84‐86, 356.

[40] T. Noritake , M. Aoki , S. Towata , Y. Seno , Y. Hirose , E. Nishibori , M. Takata , M. Sakata , Appl. Phys. Lett. 2002, 81, 2008.

[41] R. Yu , P. K. Lam , Phys. Rev. B 1988, 37, 8730. 10.1103/physrevb.37.8730 9944237

[42] I. Baraille , C. Pouchan , M. Causa , C. Pisani , Chem. Phys. 1994, 179, 39.

[43] J.‐H. Ye , Y.‐J. Zhao , Y.‐X. Fang , H.‐J. Lin , L. Bai , J.‐J. Tang , Int. J. Hydrogen Energy 2019, 44, 4897.

[44] R. Bader , Atoms in Molecules: A Quantum Theory, International series of monographs on chemistry, Clarendon Press, London 1990.

[45] G. Henkelman , A. Arnaldsson , H. Jónsson , Comp. Mat. Sci. 2006, 36, 354.

[46] B. Rösch , S. Harder , Chem. Commun. 2021, 57, 9354. 10.1039/d1cc04147a 34528959

[47] P. Sprunger , E. Plummer , Chem. Phys. Lett. 1991, 187, 559.

[48] E. Billeter , Z. Łodziana , A. Borgschulte , J. Phys. Chem. C 2021, 125, 25339. 10.1021/acs.jpcc.1c08635 PMC8607499 34824662

[49] W. S. M. Werner , J. Surf. Anal. 2005, 12, 127.

[50] R. F. Egerton , Electron Energy‐Loss Spectroscopy in the Electron Microscope, 3 edition, Springer Science+Business Media, Berlin 2011.

[51] S. Tanuma , C. J. Powell , D. R. Penn , Surf. Interface Anal. 2011, 43, 689.

[52] Z. X. He , W. Pong , Phys. Scr. 1990, 41, 930.

[53] A. Surrey , L. Schultz , B. Rellinghaus , Adv. Struct. Chem. Imaging 2016, 2, 1.

[54] B. Paik , A. Walton , V. Mann , D. Book , I. P. Jones , I. R. Harris , Appl. Phys. Lett. 2012, 100, 193902.

[55] N. J. Zaluzec , in Transmission Electron Energy Loss Spectrometry in Materials Science (Eds: M. M. Disko , C. C. Ahn , B. Fultz ), The Minerals, Metals & Materials Society, 1991.

[56] R. H. Ritchie , Phys. Rev. 1957, 106, 874.

[57] E. A. Stern , R. A. Ferrell , Phys. Rev. 1960, 120, 130.

[58] A. Voskoboinikov , N. Nakhodkin , Y. Kryn'ko , S. Kulik , P. Melnik , D. Sheka , Solid State Commun. 1994, 90, 27.

[59] J.‐T. Li , J. Parisi , Z.‐B. Wang , Y.‐K. Pu , J. Phys. D: Appl. Phys. 2014, 47, 425304.

[60] M. Alducin , S. Peter Apell , I. Zoric , A. Arnau , Phys. Rev. B 2001, 64, 125410.

[61] P. J. Feibelman , Prog. Surf. Sci. 1982, 12, 287.

[62] G. Andersson , B. Hjörvarsson , P. Isberg , Phys. Rev. B 1997, 55, 1774.

[63] H. Smithson , C. A. Marianetti , D. Morgan , A. Van der Ven , A. Predith , G. Ceder , Phys. Rev. B 2002, 66, 144107.

[64] X. Duan , R. Griessen , R. J. Wijngaarden , S. Kamin , N. Liu , Phys. Rev. Mater. 2018, 2, 085802.

[65] S. T. Kelly , B. M. Clemens , J. Appl. Phys. 2010, 108, 013521.

[66] E. Billeter , S. Kazaz , A. Borgschulte , Adv. Mater. Interfaces 2022, 9, 23.

[67] S. Hao , D. S. Sholl , Appl. Phys. Lett. 2008, 93, 1.

[68] C. Zhou , Y. Gao , R. C. Bowman , J. Zhang , H. Liu , P. Sun , Z. Z. Fang , Phys. Chem. Chem. Phys. 2021, 23, 15374. 34259266 10.1039/d1cp02498a

[69] G. Kresse , J. Furthmüller , Phys. Rev. B 1996, 54, 11169. 10.1103/physrevb.54.11169 9984901

[70] G. Kresse , J. Furthmüller , Comp. Mat. Sci. 1996, 6, 15.

[71] G. Kresse , D. Joubert , Phys. Rev. B 1999, 59, 1758.

[72] P. E. Blöchl , Phys. Rev. B 1994, 50, 17953. 10.1103/physrevb.50.17953 9976227

[73] J. P. Perdew , K. Burke , M. Ernzerhof , Phys. Rev. Lett. 1996, 77, 3865. 10062328 10.1103/PhysRevLett.77.3865

[74] G. Henkelman , B. P. Uberuaga , H. Jónsson , J. Chem. Phys. 2000, 113, 9901.

