# Supplementary material for: Why Hydrogen Dissociation Catalysts do not Work for Hydrogenation of Magnesium
Source: Adv Sci (Weinh). 2023 Dec 9;11(7):2304603. doi: 10.1002/advs.202304603 (PMC10870026; doi:10.1002/advs.202304603)
Supplement: Supplementary file 1 — Supporting Information [file ADVS-11-2304603-s001.pdf]

ADVANCED SCIENCE
Open Access

Supporting Information

for *Adv. Sci.*, DOI 10.1002/advs.202304603

Why Hydrogen Dissociation Catalysts do not Work for Hydrogenation of Magnesium

Selim Kazaz, Emanuel Billeter, Filippo Longo, Andreas Borgschulte and Zbigniew Łodziana**

Supporting Information to "Why Hydrogen Dissociation Catalysts do not Work for Hydrogenation of Magnesium"

October 13, 2023

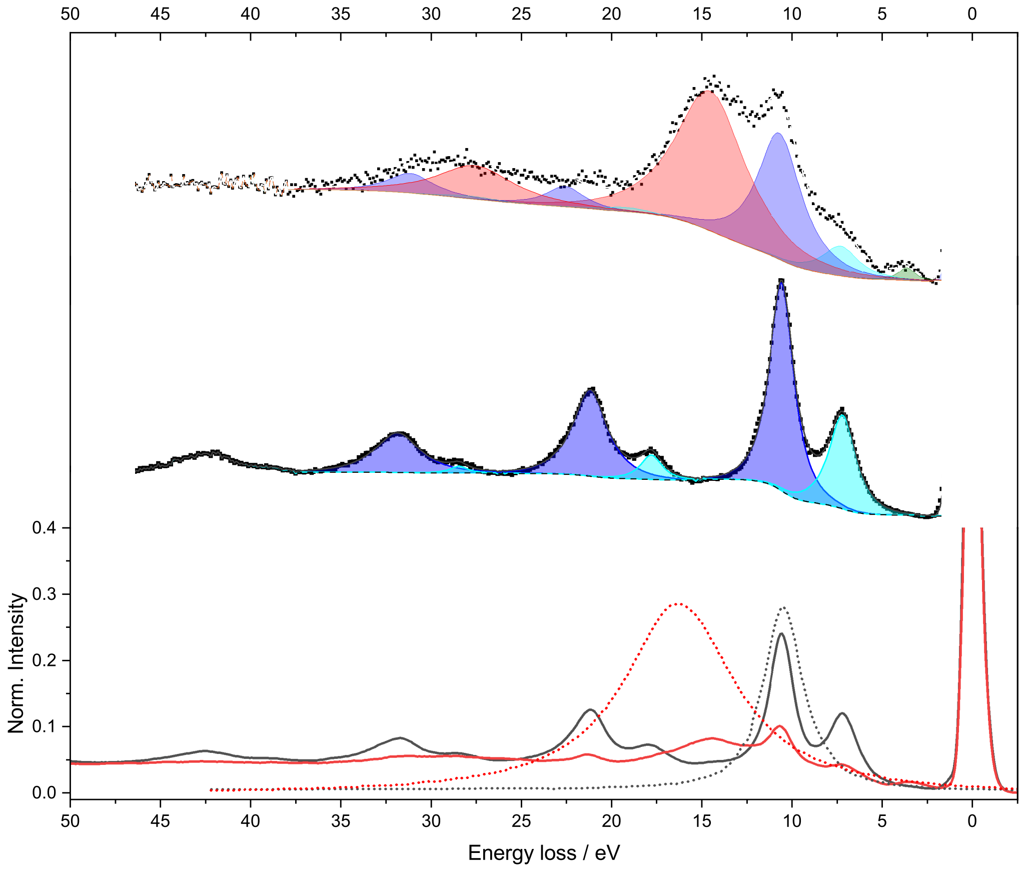

Figure S1: Bottom: REELS of Mg before (black) and after hydrogenation (red), recorded at an incident energy of $E_i = 2$ keV. Bulk data (dotted lines) from [1]. The evolving MgH_2 peak at 14.6 ± 0.1 eV is in good agreement with literature values (14.6 ± 0.2 eV [2], 14.8 ± 0.1 eV [3], 14.2 ± 0.5 eV [4]). Fits of the pristine (middle) and hydrogenated Mg films (top) using CasaXPS, recorded at an incident energy of $E_i = 2.5$ keV.

Estimation of the Mg layer thickness for the various samples is based on the assumption of a homogeneous layer with a defined composition and was calculated using the following equation [5]:

$$d(nm) = \lambda_{\mathrm{Mg}} \cdot \sin\theta \cdot \ln\left(\frac{N_{\mathrm{SiO_2}} \cdot \lambda_{\mathrm{SiO_2}} \cdot I_{\mathrm{Mg}}}{N_{\mathrm{Mg}} \cdot \lambda_{\mathrm{Mg}} \cdot I_{\mathrm{SiO_2}}} + 1\right) \tag{1}$$

with θ the photoelectron take-off angle, λ_{Mg} the IMFP for the Mg, $\lambda_{\mathrm{SiO_2}}$ the IMFP for SiO_2, N_{Mg} and $N_{\mathrm{SiO_2}}$ the volume density of Mg and SiO_2 in the matrix, respectively, I_{Mg} the intensity of the area arising from Mg and $I_{\mathrm{SiO_2}}$ the intensity of the area arising from SiO_2.

Volume density of Mg and SiO_2 atoms were calculated using the following equations:

$$N = \frac{\rho \cdot C}{M} \tag{2}$$

with ρ the bulk density of the species, C the relative concentration in at% of the species and M the related atomic/molecular weight. Table T1 reports the values employed in the two previous equations, while Table T2 reports the calculated Mg thickness for both XPS and HAXPES.

	ρ (g/cm^{-}3)	VE n°	M (g/mol)	IMFP$_{XPS}$ (Å)	IMFP$_{HAXPES}$ (Å)
Mg	1.74	2	24.31	38.21	91.09
SiO_2	2.65	16	60.08	36.43	80.76

Table T1: Bulk density (ρ), valence electrons (VE), atomic/molecular weight (M) for Mg and SiO_2 used to calculate the corresponding IMFP with QUASES-IMFP-TPP2M software [6], both for XPS and HAXPES.

Sample	d (nm) XPS	d (nm) HAXPES
Mg5	4.6	9.0
Mg10	ND	17.1
Mg20	ND	ND

Table T2: Calculated magnesium layer thickness for XPS and HAXPES after 5, 10 and 20 minutes sputtering.

Calculations details

An extensive accuracy assessment was done prior to the actual calculations, it focused on appropriate description of the electronic structure via choice of Mg valence configuration, sufficient accuracy and feasibility of the calculations. The calculated lattice parameters for magnesium are: $a = 3.194$ Å, $c = 5.163$ Å(valence configuration $3s^2$); $a = 3.197$ Å, $c = 5.166$ Å($2p^6 3s^2$); $a = 3.187$ Å, $c = 5.152$ Å($2s^2 2p^6 3s^2$, the energy cutoff 620 eV). They can be compared to the experimental lattice parameters at room temperature $a = 3.2085$ Å $c = 5.2106$ Å [7]. The corresponding data for MgH_2 is shown in Table T3.

	$3s^2$	$2p^6 3s^2$	$2s^2 2p^6 3s^2$	$3s^2$ (VdW)	$2p^6 3s^2$ (VdW)	$2s^2 2p^6 3s^2$ (VdW)
Mg	+1.60	+1.62	+1.63	+1.62	+1.64	+1.64
H	-0.80	-0.81	-0.815	-0.81	-0.82	-0.82
a	4.510 Å	4.510 Å	4.509 Å	4.540 Å	4.538 Å	4.556 Å
c	3.013 Å	3.013 Å	3.013 Å	3.022 Å	3.022 Å	3.018 Å

Table T3: Bader charges on ions in e for MgH_2 and the lattice parameters calculated with different representations of the Mg valence electrons in PAW potentials. VdW stands for calculations performed with dispersive interactions according to method of [8]. For Mg with electronic configuration $2s^2 2p^6 3s^2$ the energy cutoff was 620 eV. The experimental MgH_2 lattice parameters at room temperature are $a = 4.5168$ Å $c = 3.0205$ Å [9].

The lattice parameters are in mutual agreement within 0.04 Åwith the significant difference originating from incorporation of weak dispersive interactions. One might also notice a systematic, small, increase of the charge transfer, this shall be expected [10] but it is rarely noticed in the literature. Taking into account the computational cost this interaction was not explicitly included in further calculations.

Spin polarized calculations for MgH_2, $MgH_{1.5}$, MgH_1, $MgH_{0.5}$ provide no difference with respect to the ground state energy or structural parameters. For that reasons in calculations Mg was represented by $2p^6 3s^2$ valence electronic configuration, the spin polarization was not used. However for each relevant system a selected configuration was recalculated with spin polarization that reveal no meaningful differences in charge distribution, energies or structure.

For the surface calculations (0001), (10-10), (10-11) surface were cleaved for Mg; (100), (101), and (110) for MgH_2 with appropriate lattice parameters above. A symmetric slab was created with sufficient thickness to avoid mutual interactions between the surface, e.g. Fig. S2.

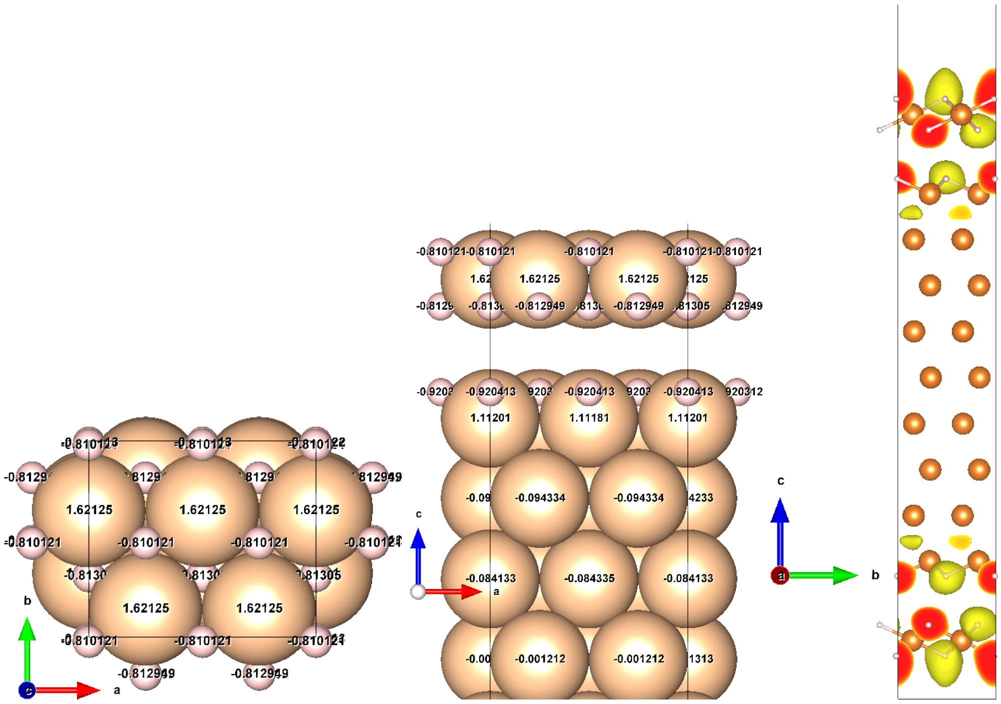

Figure S2: The Bader charges on atoms and electron localization function for Mg(0001) surface with 3 ML of hydrogen adsorbed (left and center). The electron localization function for the calculation of symmetric setup with 11 atomic layers of Mg (right).

The symmetric slab was used for all systems with metallic Mg, for MgH_2 the bottom of the slab was frozen and dipole correction applied and for the model single layer vacuum was 20 Å. The lateral dimensions of the slabs were 2×2 for $MgH_2(100)$, 4×2 for $MgH_2(110)$, 1×1 for $MgH_2(101)$, 2×1 for Mg(10-10), 2×1 for Mg(10-11), $2\times1R45$ for Mg(0001), and $4\times2R45$ for Mg(0001) isolated layers. The details of the charge distribution at $MgH_2(110)$ surface with 50% of hydrogen vacancies are depicted in Fig. S3 In all calculations where defects as H vacancies are present several (usually six

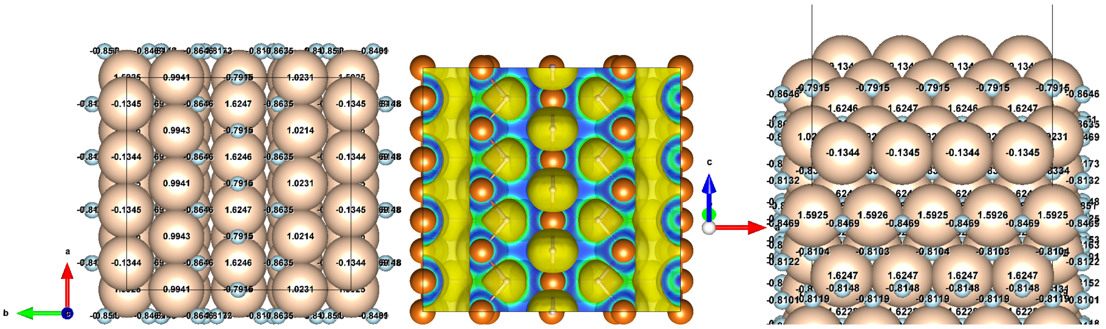

Figure S3: The Bader charges on atoms and electron localization function for the lowest energy configurations of the hydrogen vacancies at the $MgH_2(110)$ with 50% of surface hydrogen vacancies. The larger spheres are for Mg, smaller ones are for H.

to eight) independent defects configurations were considered and structure (or more, if necessary) with the lowest defect formation energy was considered for detailed analysis. An example of such calculations is depicted in Fig. S4 for vacancies on MgH_2. A variety of H vacancy distributions at the surface was considered. This include a layer by layer H stripping and formation of H deficient clusters, regions where metallic Mg is present. The formation enthalpy of H vacancy varies from 0.58 eV/VH for 25% H vacancy density through 0.57 eV/VH and 0.54 eV/VH for 37.5% and 50% that is only weakly dependent on their density. For each vacancy density the configurations with metallic Mg are more stable than those with isolated Mg atoms by 0.11 eV - 0.15 eV S4. For 1ML of H vacancies the stability of metallic Mg overlayer is slightly lower (by 0.02 eV) than two dimensional Mg structure extending to the second subsurface atoms. This indicates that other than layer by layer decomposition mechanisms of MgH_2 may exist.

Similar example for analysis of the defects distribution on the model of isolated Mg(0001) slab

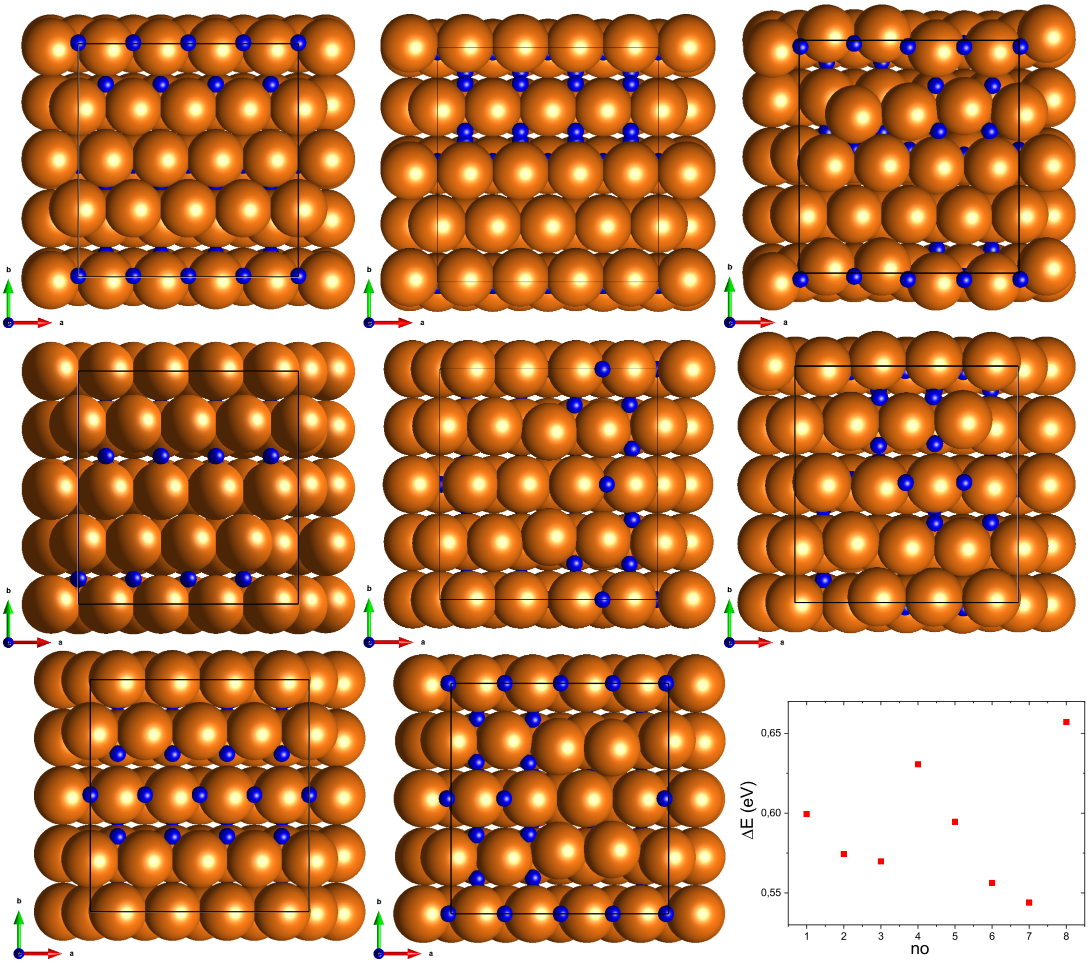

Figure S4: The configurations of the hydrogen vacancies for the MgH_2(110) with 50% of surface hydrogen vacancies. The formation energy per vacancy is shown in bottom right corner, large spheres are for Mg, small ones for H.

with stoichiometry $MgH_{1.5}$ is presented in Fig. S5.

In Fig. S6 the electron localization function for different concentrations of hydrogen vacancies at the surface is shown. In the right hand side section of this figure the lowest energy configurations are present and for higher vacancy concentrations metallic Mg (continuous regions) are visible. This indicated that even locally the intermediate MgH_x stoichiometries are less favorable than the interface between Mg and MgH_2.

The charge analysis was performed on the density grids with spacing lower than 0.05 Å, the same grid was used for calculations of the electron localization functions. As the charge strongly depends on coordination, distances between atoms in models with sufficient number of defects charges can differ slightly at host/defect interface. This result in discreet distribution of charges that differ by a few %. In order to make analysis possible the charges were stored in the histograms with the bin size 0.2 e for Mg and 0.02 e for H. This results in broadening for simpler cases where the charge on atoms is uniquely defined, however for the consistency the broadening is applied for all cases. The detailed charge distribution on atoms can be inspected in Figs. S2,S3,S13,S14.

The Fig S7b depicts the Mg charge in the model system of a single the Mg(0001) layer. Such system corresponds to sub-mono layer H adsorption and our choice of large supercell allows tracking of H configuration in such layer, see below. From the charge transfer perspective it can be noticed that only for MgH_x, $x > 1.25$ the oxidation state of Mg reaches +1.6e. For lower amounts of adsorbed hydrogen the charge state of Mg is combination of 0e, +1e, +2e. The MgH_2 (110) surface reveals the charge of +1.62e at the first layer the Mg, Fig. S7c. For the surface with 50% of H vacancies three distinct oxidation states of Mg are present, while for the intermediate number of vacancies the combinations of +1e and +2e are observed.

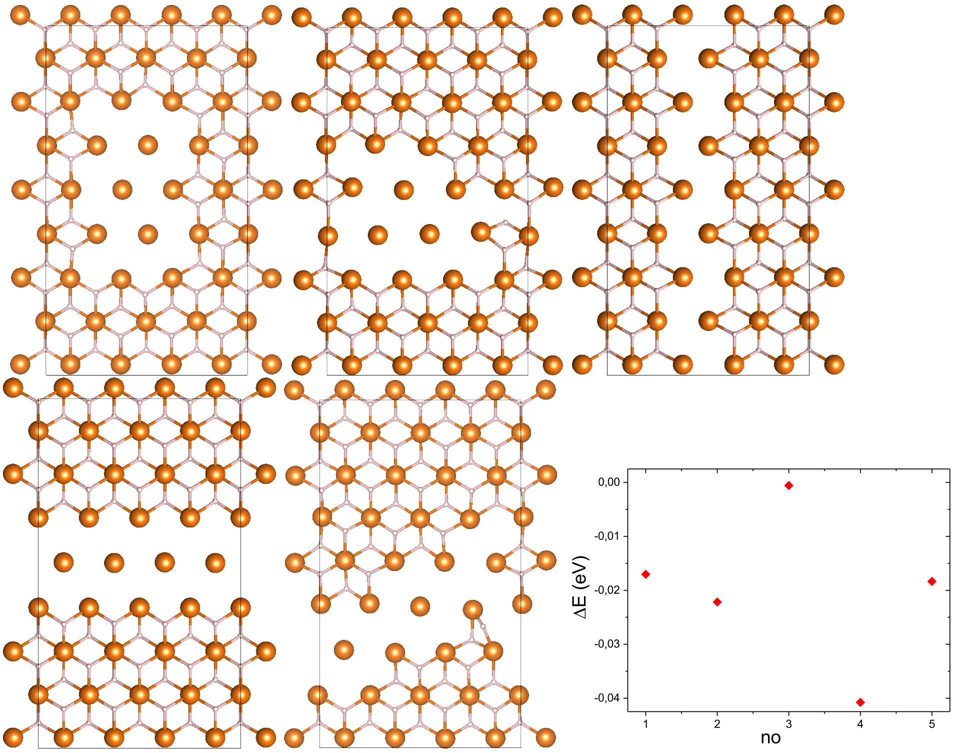

Figure S5: The configurations considered for hydrogen distribution in the model system with $MgH_{1.5}$ stoichiometry. The formation enthalpy of the systems is shown in bottom right corner. The configuration with the lowest energy was a subject for a detailed hydrogen migration pathway and the charge distribution analysis as detailed in Fig. S13.

The formation enthalpy of hydrogen interstitial in the bulk Mg is presented in Fig. S8 (left) together with the Mg charge distribution in the vicinity of these interstitial.

Hydrogen agglomerates that are smaller than 20 atoms can accommodate the lattice strain of Mg. Preferred location of hydrogen is at the tetrahedral coordination. For smallest isolated clusters Mg^{+2} is not present thus they are unstable. The electron localization function for selected interstitial configurations is depicted in Fig. S9.

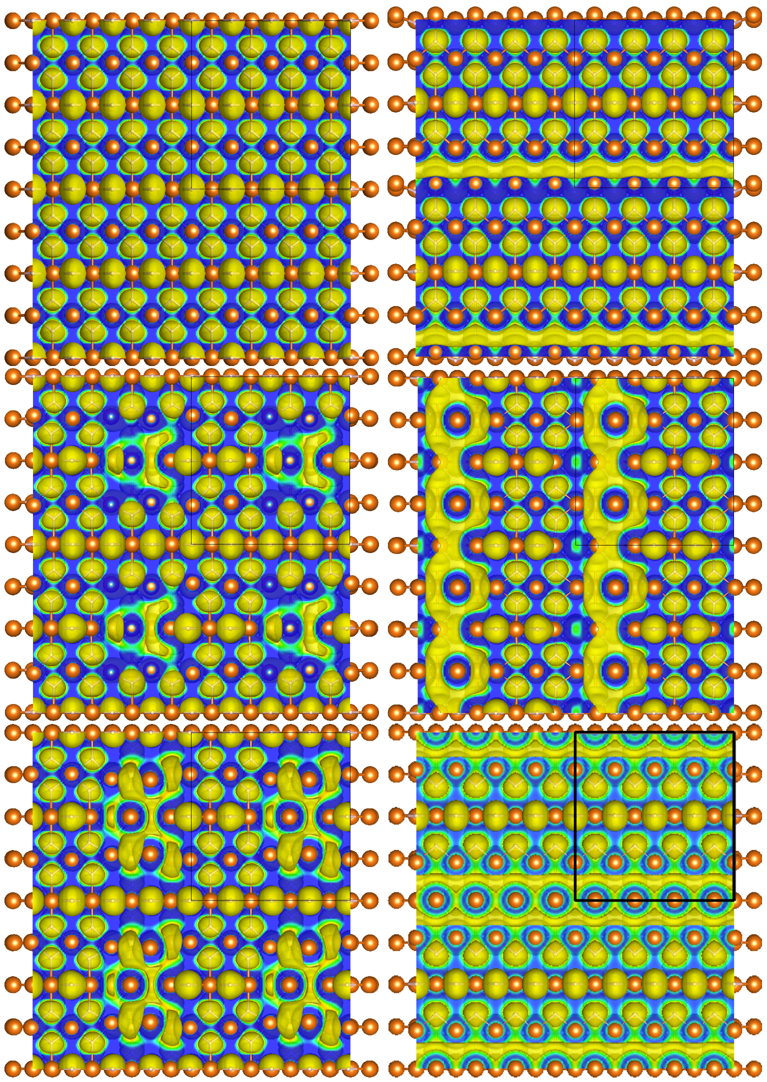

Figure S6: The electron localization for MgH_2(110) surface (upper left); this surface with 25% H vacancy (upper right). The middle panel is for 37.5% H vacancy, the bottom one for 50%. On the left side the most stable configuration with metallic Mg is shown, the right without metallic Mg is less stable by 0.1 - 0.2 eV. The yellow color correspond to the electron localization, either on H or the conduction bands of Mg.

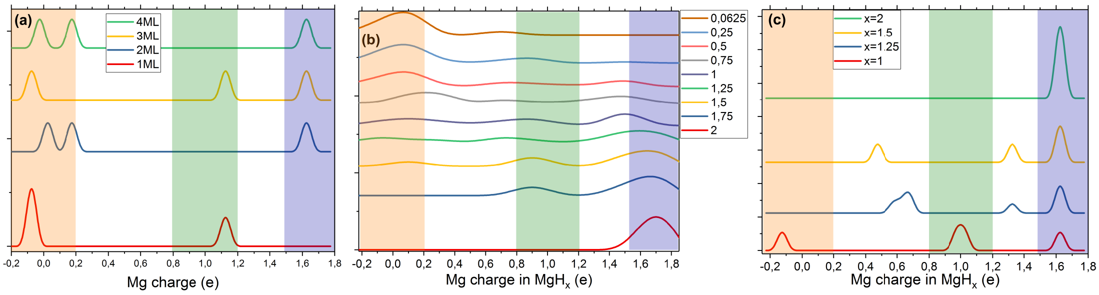

Figure S7: The calculated Bader charge on Mg for H adsorbed on Mg(0001) surface with coverage from 1 ML to 4 ML (a), in the model system of the single MgH_2 layer of different stoichiometry (b), and on H deficient MgH_2(110) surface, where the stoichiometry refers to the topmost layer (c). The shaded orange, green and blue regions refer to formal oxidation states of Mg 0, +1, and +2 respectively.

The activation energies for hydrogen migration in the model system is presented in Fig. S10, S11, S12, S13, S14. For each case the vibrational frequencies and the atomic displacements related to the imaginary mode at the transition state are deposited in XXXXX.

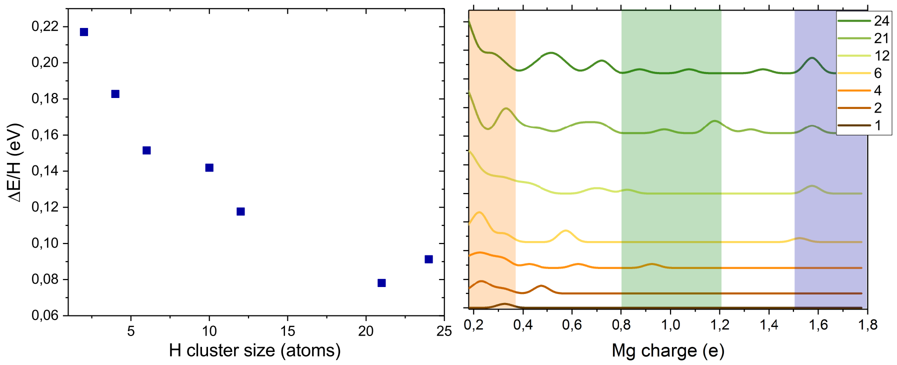

Figure S8: The energy of H cluster formation in the bulk Mg. The calculations are for $(4 \times 4 \times 3R45)$ supercell that gives the nominal stoichiometry $MgH_{0.167}$ for the cluster of 24 atoms. The calculations are performed for the fixed volume, thus the increase of the energy is related to lattice strains that become dominant above 21 atom H cluster. In the right panel the distribution of the ionic charges on Mg for different cluster sizes (charge ~ 0 is not shown for clarity).

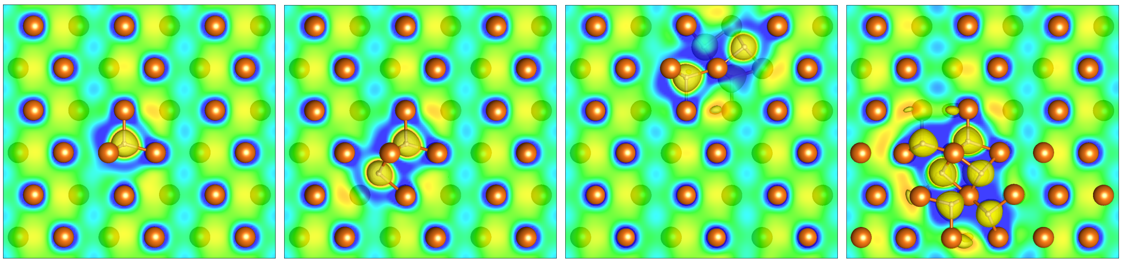

Figure S9: The electron localization for H clusters in Mg for 1, 2, 4, and 12 atoms hydrogen cluster in magnesium. The yellow color stands for localized electrons. The conduction band carriers are visible in the metallic Mg region, ionic charge on H^- around hydrogen and for larger cluster the charge accumulation due to polarization effects is also visible as denser yellow regions for two rightmost plots.

An example of the process of H_2 dissociation on MgH_2 layer, the activation energy is relatively large, however does not exclude such process S15.

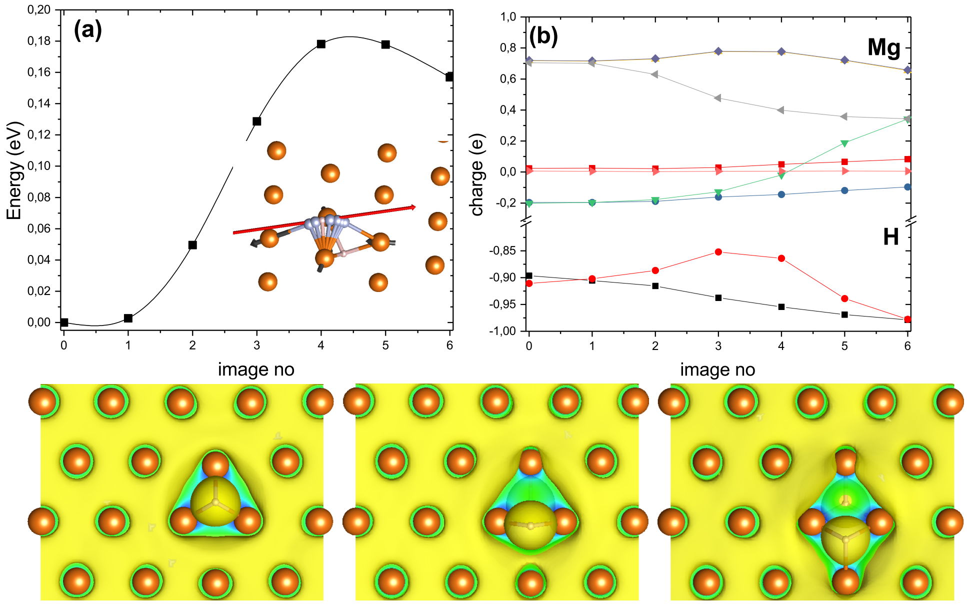

Figure S10: The calculated energy barrier for H diffusion on Mg(0001) layer with stoichiometry $MgH_{0.0625}$ (a) and the charge distribution on H and adjacent Mg atoms (b). The low energy barrier is correlated with small charge modifications on Mg at the transition state. The transition path related to the H hoping is shown as an insert in together with the directions of relevant atomic displacements related to the imaginary mode (a), orange spheres are for Mg, small gray for H. The process presented corresponds to the transition of H form the lowest energy configurations of 2 H atoms adsorbed on the opposites trihedral sites to the adsorption of 2 H atoms on the nearest sites. The bottom panel shows the electron localization for the initial, transition and final state (left to right) as well as the atomic displacements related to the imaginary mode at the transition state.

References

[1] A. Surrey, L. Schultz, B. Rellinghaus, *Advanced Structural and Chemical Imaging* **2016**, *2*, 1 1.

[2] Z. X. He, W. Pong, *Phys. Scr.* **1990**, *41* 930.

[3] B. Paik, A. Walton, V. Mann, D. Book, I. P. Jones, I. R. Harris, *Appl. Phys. Lett.* **2012**, *100*, 19 193902.

[4] N. J. Zaluzec, In *Proceedings of TMS 1991 Annual Meeting.* **1991**.

[5] A. Larsson, G. D'Acunto, M. Vorobyova, G. Abbondanza, U. Lienert, Z. Hegedüs, A. Preobrajenski, L. R. Merte, J. Eidhagen, A. Delblanc, J. Pan, E. Lundgren, *Journal of Alloys and Compounds* **2022**, *895* 162657.

[6] S. Tanuma, C. J. Powell, D. R. Penn, *Surf. Interf. Anal.* **1994**, *21* 165.

[7] E. A. Owen, L. Pickup, I. O. Roberts, M. Sc., *Zeitschrift für Kristallographie - Crystalline Materials* **1935**, *91* 70.

[8] M. Dion, H. Rydberg, E. Schröder, D. Langreth, B. Lundqvist, *Phys. Rev. Lett.* **2004**, *92* 246401.

[9] F. H. Ellinger, C. E. J. Holley, B. B. McInteer, D. Pavone, R. M. Potter, E. Staritzky, W. H. Zachariasen, *J. Am. Chem. Soc.* **1955**, *77* 2647.

[10] N. Ferri, A. Ambrosetti, A. Tkatchenko, *Phys. Rev. Mater.* **2017**, *1* 026003.

[11] J. Isidorsson, M. E. Giebels, R. Griessen, H. Arwin, *Phys. Rev. B - Condens. Matter Mater. Phys.* **2003**, *68*, 11 1.

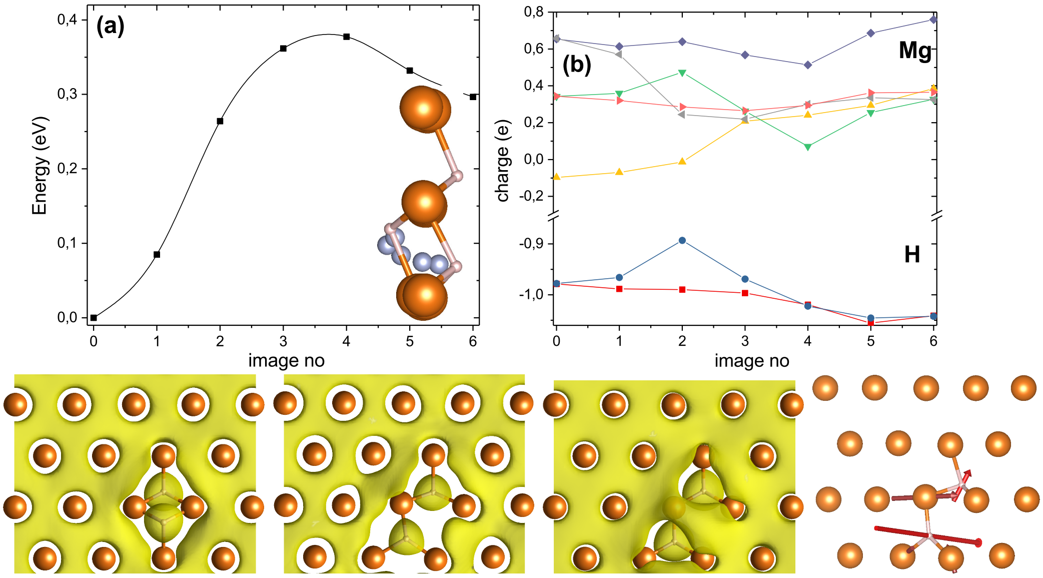

Figure S11: The calculated energy barrier for H diffusion through Mg(0001) layer with stoichiometry $MgH_{0.0625}$ (a) and the charge distribution on H and adjacent Mg atoms (b). The low energy barrier is correlated with small charge modifications on Mg at the transition state. The transition path related to the H hoping is shown as an insert in (a), orange spheres are for Mg, small gray for H. The process presented corresponds to the transition 2 H atoms adsorbed on the nearest sites to the opposite sites. The bottom panel shows the electron localization for the initial, transition and final state (left to right) as well as the atomic displacements related to the imaginary mode at the transition state.

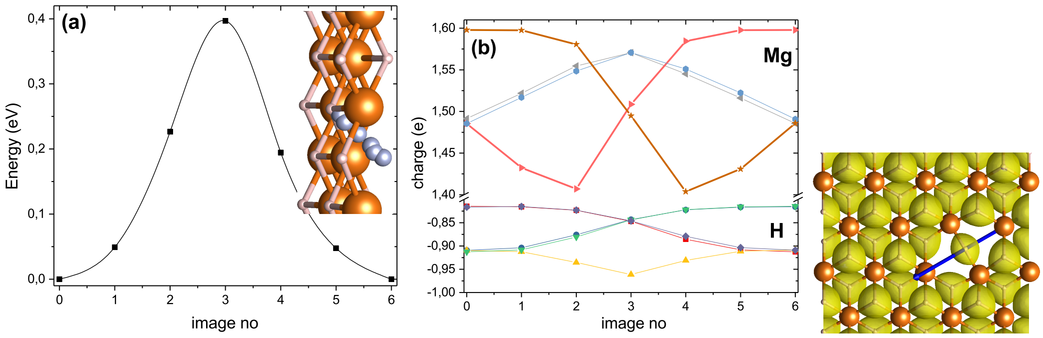

Figure S12: The calculated energy barrier for vacancy H diffusion through fully hydrateted Mg(0001) layer with stoichiometry $MgH_{1.969}$ (a) and the charge distribution on H and adjacent Mg atoms (b). The low energy barrier is correlated with small charge modifications on Mg at the transition state. The transition path related to the H hoping is shown as an insert in (a), orange spheres are for Mg, small gray for H. The process presented corresponds to the transition the H vacancy between the opposite sides of the layer. The atomic displacements and electron localization at the transition state is shown in the right.

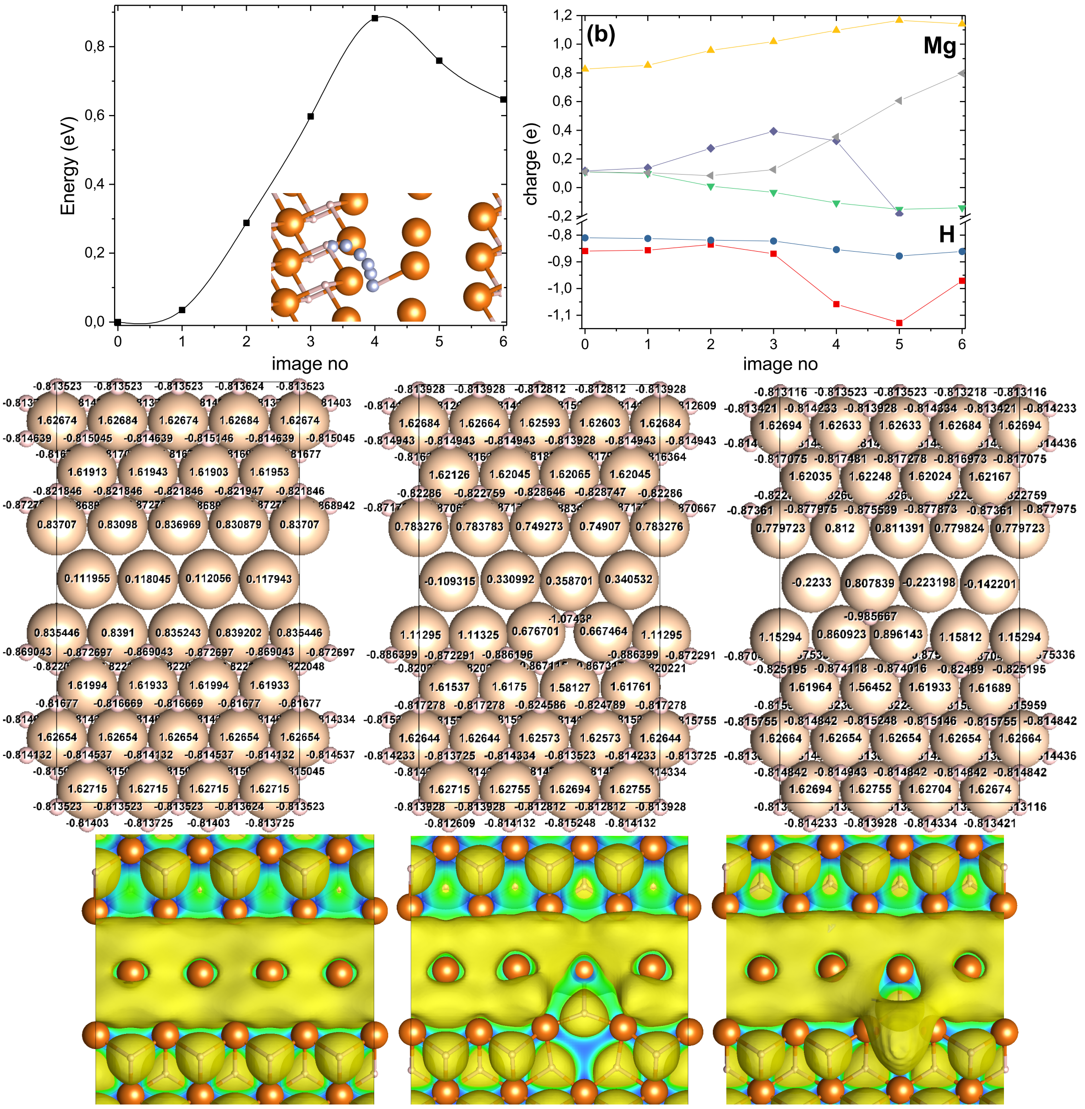

Figure S13: The calculated energy barrier for H atom diffusion from hydrated cluster to metallic part of the Mg(0001) layer layer with stoichiometry $MgH_{1.5}$. The transition path related to the H hoping is shown as an insert in, orange spheres are for Mg, small gray and pink are for H, white is for H passing through. Bottom panels show the Bader charges on atoms and electron localization function for the process oh hydrogen atom hoping from the equilibrium position (left) through the transition state (center) to the metastable location on the opposite side (right) in the model system with $MgH_{1.5}$ stoichiometry. The corresponding electron localization is shown below.

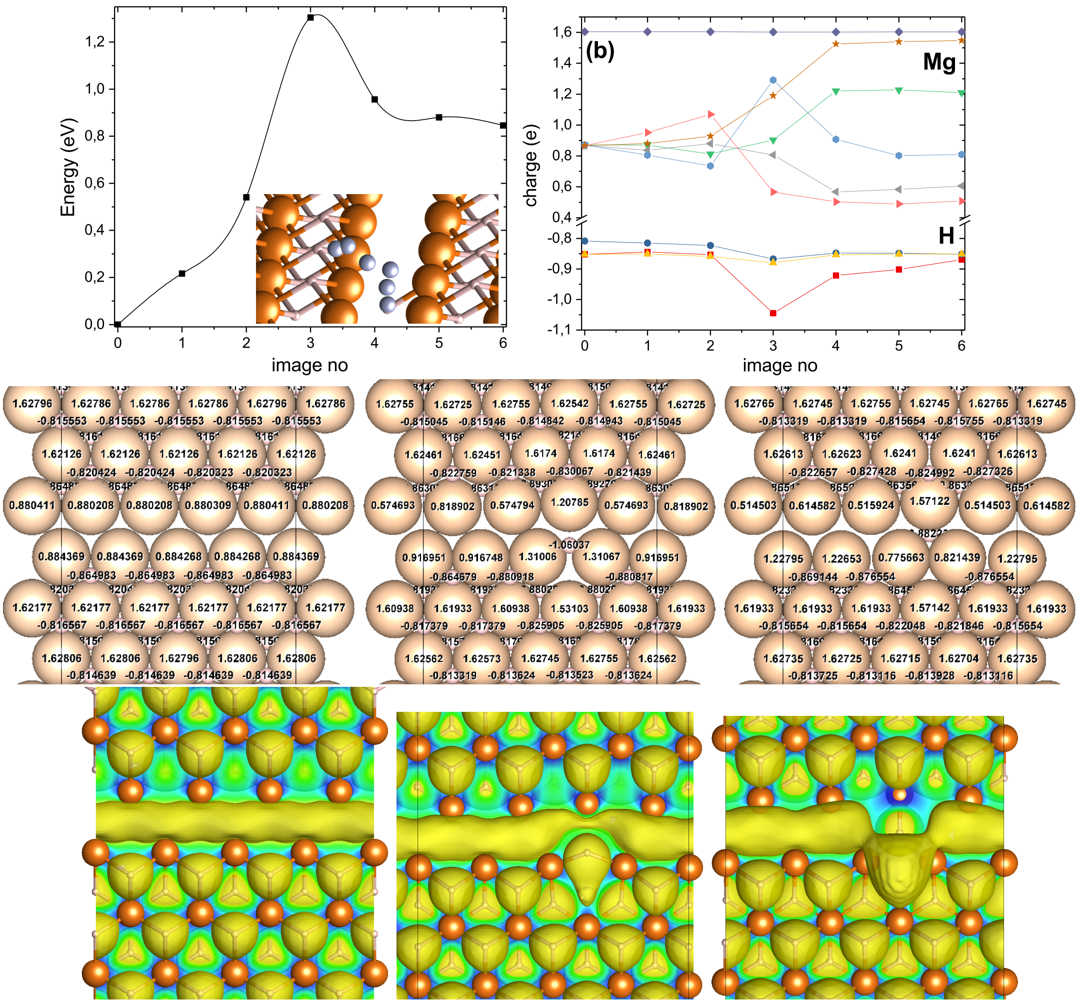

Figure S14: The calculated energy barrier for H atom diffusion from hydrated cluster to the cluster boundary region in the Mg(0001) layer layer with stoichiometry $MgH_{1.75}$. The transition path related to the H hoping is shown as an insert in, orange spheres are for Mg, small gray and pink are for H, white is for H passing through. Bottom panels show the Bader charges on atoms and electron localization function for the process oh hydrogen atom hoping from the equilibrium position (left) through the transition state (center) to the metastable location on the opposite side (right) in the model system with $MgH_{1.5}$ stoichiometry. The corresponding electron localization is shown below.

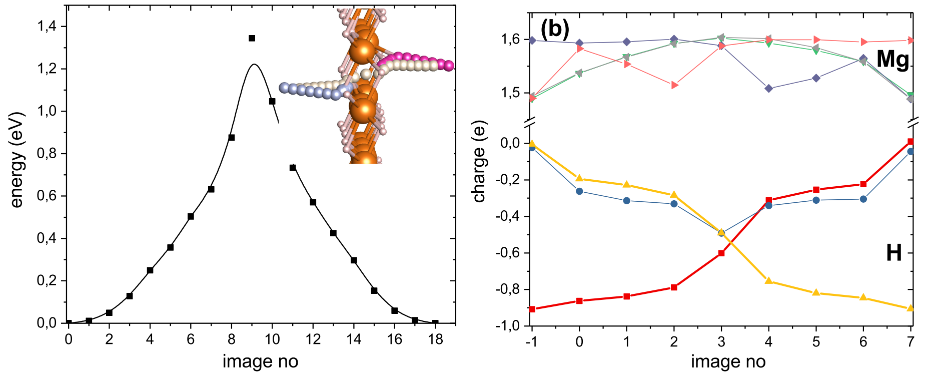

Figure S15: The calculated energy barrier for H_2 dissociation and diffusion through fully hydrateted Mg(0001) layer. The energy barrier is related H_2 dissociation over H vacancy of MgH_2 layer. The transition path related to the H hoping is shown as an insert in, orange spheres are for Mg, small gray and pink are for H, white is for H passing through. The process presented corresponds to dissociation of H_2 at one side of the layer, pass though of H atom and H_2 association on the other side.

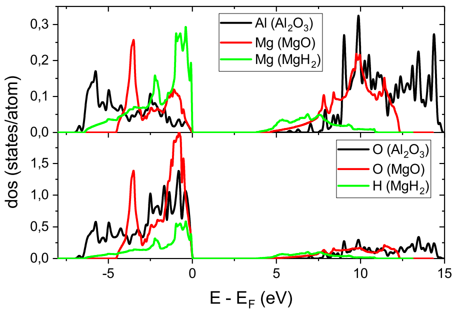

Figure S16: The density of states projected on metal cations (upper panel) and anions in lower panel for Al_2O_3, MgO, and MgH_2 [11, 3].
